# Extracellular microRNAs profile in human follicular fluid and IVF outcomes

**DOI:** 10.1038/s41598-018-35379-3

**Published:** 2018-11-19

**Authors:** Rosie M. Martinez, Liming Liang, Catherine Racowsky, Laura Dioni, Abdallah Mansur, Michal Adir, Valentina Bollati, Andrea A. Baccarelli, Russ Hauser, Ronit Machtinger

**Affiliations:** 1 Department of Environmental Health, Harvard T.H. Chan School of Public Health, Boston, Massachusetts 02115 USA; 2 Laboratory of Precision Environmental Biosciences, Department of Environmental Health Sciences, Columbia Mailman School of Public Health, New York, New York 10032 USA; 3 Department of Biostatistics, Harvard T.H. Chan School of Public Health, Boston, Massachusetts 02115 USA; 4 Department of Obstetrics, Gynecology and Reproductive Biology, Brigham and Women’s Hospital, Harvard Medical School, Boston, Massachusetts 02115 USA; 5 EPIGET - Epidemiology, Epigenetics and Toxicology Lab, Department of Clinical Sciences and Community Health, University of Milan, 20122 Milano, Italy; 6 Department of Obstetrics and Gynecology, Sheba Medical Center, Ramat-Gan, 52561 Israel; 7 Sackler School of Medicine, Tel-Aviv University, Tel-Aviv, Israel

## Abstract

Encapsulated microRNAs (i.e., miRNAs within the extracellular vesicles, i.e., EV-miRNAs) have been detected in follicular fluid in both animal and human studies and different profiles have been associated with IVF cycle characteristics. However, limited studies to date have investigated other IVF outcomes, including fertilization status and embryo quality on day three”. In this cohort, we performed a cross-sectional analysis on 126 women who contributed follicular fluid from a single follicle during a single IVF cycle. One hundred and ninety-two EV-miRNAs were assessed by univariable fold-change and multivariable logistic regression analyses. Hsa-miR-92a and hsa-miR-130b, were over-expressed in follicular fluid samples from oocytes that failed to fertilize compared to those that were normally fertilized. Additionally, hsa-miR-888 was over-expressed and hsa-miR-214 and hsa-miR-454 were under-expressed in samples that resulted in impaired day-3 embryo quality compared to top-quality day-3 embryos. After adjusting for confounders as BMI, smoking and total motile sperm, associations of these EV-miRNAs remained significant. In-silico KEGG pathway analyses assigned the identified EV-miRNAs to pathways of follicular growth and development, cellular signaling, oocyte meiosis, and ovarian function. Our findings suggest that EV-miRNAs may play a role in pathways of ovarian function and follicle development, which could be essential for understanding the molecular mechanisms that could lead to a successful pregnancy and birth.

## Introduction

Follicular fluid is the critical microenvironment for the development of oocytes^1,2^ and contains a mixture of proteins, metabolites, ions, plasma components, and various molecules including non-coding RNAs. Bidirectional cross-talk between the oocyte and the granulosa cells that line the ovarian follicle is necessary for oocyte competence, fertilization, and ultimately embryo development^3,4^.

Recent reports have demonstrated that extracellular vesicles (EVs), which include exosomes, microvesicles and other similar membrane-bound vesicles, can be up-taken *in vivo* and *in vitro* by follicular cells, suggesting a possible signaling role within the ovarian follicle^5–7^. EVs have been detected in almost all body fluids including blood, urine, breast milk, semen and follicular fluid^8^. They are of nanosize, ranging between 30 to 1000 nm in diameter, and can promote intracellular communication by being a vehicle that can carry proteins, messenger RNAs (mRNAs), and non-coding RNAs as microRNAs (miRNAs)^9^. MiRNAs are short non-coding RNA molecules (approximately 22 nucleotides in length) that can influence gene expression by regulating essential cellular processes including growth, differentiation, and apoptosis^10–12^. They have been associated with both normal and pathological conditions^13^ and can be important for follicular signaling^14,15^.

These EV-encapsulated miRNAs (EV-miRNAs) make up a subset of total extracellular RNA. EV-miRNAs are extremely stable and have shown resistance to degradation, compared to free-floating microRNAs, most likely due to their enclosed state^16^. Recent animal and human studies have found that EV-miRNAs in follicular fluid are associated with targets that regulate insulin, epidermal growth factor receptor (ErbB), mitogen-activated protein kinase (MAPK), Wnt signaling pathways, and transforming growth factor beta (TGFB)^7,17–20^.

In a previous explorative study, we identified EV-miRNAs present in human follicular fluid containing mature oocytes and tried to characterize miRNAs that were associated with fertilization and embryo quality^21^. Following our exploratory results, we expanded our study to a large cohort of different women undergoing IVF and isolated EVs by ultra-centrifugation, which has become the gold standard for EV extraction. The aim of our study was to assess whether EV-miRNAs from follicular fluid can serve as biomarkers for fertilization status and day 3 embryo quality.

## Results

### Participants

**Figure 1:**
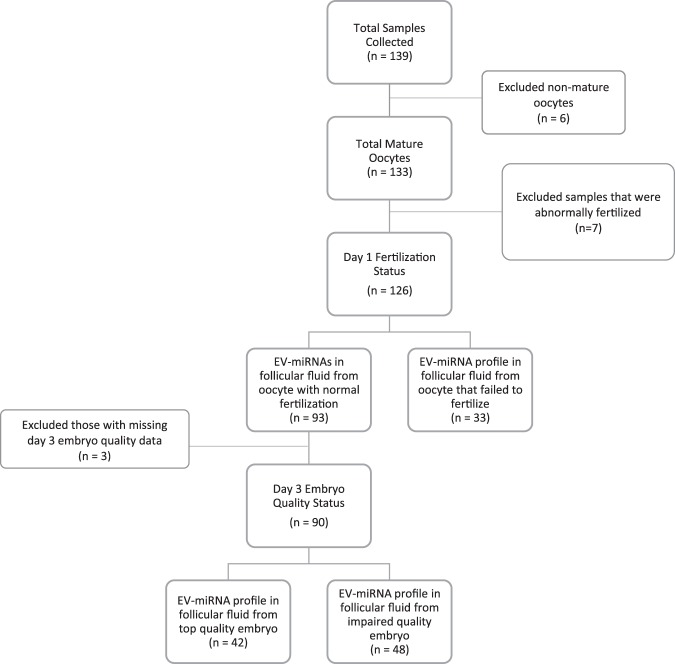
Flow chart of analyses. Examining the flow of analyses in this study and how exclusion criteria apply.

We collected 139 follicular fluid samples but excluded 6 follicular fluid samples from the analyses because either the oocytes were not mature (n = 4) or the number of oocytes retrieved was greater than 25 (n = 2); the latter were excluded due to the possibility that the two patients involved may have had PCOS despite not being classified *a priori* as such (in both cases high numbers of oocytes were retrieved [27 and 49] and low doses of gonadotropins were used). We further restricted our samples to exclude those patients associated with abnormal fertilization or atretic oocytes on the day after retrieval (n = 7). The final number of patients contributing follicular fluid samples was 126 (Fig. 1). The women were 31.0 [28.9,33.7] (median [interquartile range [IQR]]) years of age, had a median BMI of 22.9 kg/m^2^ [20.1, 26.4], had undergone 1.0 IVF attempts [1, 2] (range 1–6 cycles), had a median of 9 oocytes [6, 13] (range 2–24) retrieved during the IVF cycle, and had a median total motile sperm count of 29.4 million/ml [0.79, 121.8] (Table 1). Additionally, the majority of our population were non-smokers (80%) and used the ICSI (92%) as a method of fertilization.

**Table 1:** Comparing descriptive statistics of the total study population to study participants included.

		Entire Population	Included Population	p-value^b^
N		133	126	
Age, median (IQR)		31.0 (28.9, 33.7)	31.0 (28.9, 33.7)	0.92
BMI, median (IQR)		22.9 (20.0, 26.4)	22.9 (20.1, 26.4)	0.95
Smoking Status, n (%)	Non-smoker	106 (80%)	100 (79%)	1
	Smoker	27 (20%)	26 (21%)	
Number of IVF attempts, median (IQR)		1 (1, 2)	1 (1, 2)	0.96
Number of oocytes retrieved, median (IQR)		9 (6, 12)	9 (6, 13)	0.79
Total Motile Sperm (million/mL), median (IQR)		28.80 (0.78, 120.0)	29.4 (0.79, 121.8)	0.91
Fertility Status, n (%)	Fertile	58 (44%)	55 (44%)	1
	Infertile	75 (56%)	71 (56%)	
Fertility Diagnosis, n (%)	PGD	57 (43%)	55 (44%)	1
	Male Factor	54 (41%)	50 (40%)	
	Unexplained	20 (15%)	19 (15%)	
	Other^a^	2 (1%)	2 (1%)	
Batch, n (%)	Batch 1	75 (56%)	69 (55%)	0.89
	Batch 2	58 (44%)	57 (45%)	
Fertilization Method	ICSI	122 (92%)	115 (91%)	1
	Insemination	11 (8%)	11 (9%)	
Fertilization Status, n (%)	Normal	93 (70%)	93 (75%)	0.03
	Failed Fertilization	33 (25%)	33 (25%)	
	Abnormal	7 (5%)	—	
Day 3 Quality, n (%)	Top Quality	42 (32%)	42 (33%)	0.92
	Not Top Quality	50 (38%)	48 (38%)	
	Missing	41 (30%)	36 (29%)	

Abbreviations: IVF: *In Vitro* Fertilization, IQR: inter quartile range, PGD: preimplantation genetic diagnosis, ICSI: intracytoplasmic sperm injection, BMI: body mass index.

^a^Other includes: mechanical (n = 1), and egg donor (n = 1).

^b^p-value calculated using Student’s T-test for continuous variables and Chi-Squared test for categorical variables.

### Profile of EV-miRNAs in follicular fluid containing MII oocytes

We screened for 754 EV-miRNAs and detected expression of 320 in one or more of the 126 samples analyzed. Of these, before applying global mean normalization, 22 EV-miRNAs had 100% detection in all 126 samples (Supplementary Table S1). For further analyses, we chose to examine EV-miRNAs that were detected in at least 15% of the samples resulting in 192 EV-miRNAs. Based on the histogram distribution, we found that the 15% detected mark appeared to be the cut point to separate the majority of the EV-miRNAs from the outliers (those less than 15% detected). A list of these selected EV-miRNAs with their detectable levels is provided in Table S1 (Supplementary Table S1).

### EV-miRNAs in follicular fluid and fertilization status

To examine the associations between follicular fluid EV-miRNAs and fertilization, we compared follicular fluid samples from oocytes that were normally fertilized (n = 93) to those that failed to fertilize (n = 33). We first performed unadjusted analysis using Mann-Whitney U or Student *t-*tests and calculated fold-change (FC) differences in the EV-miRNAs to compare normal vs failed fertilization. We identified 12 EV-miRNAs that were different (p ≤ 0.05) between the normal and failed to fertilize groups. Specifically, one EV-miRNA was under-expressed in the failed to fertilize group, hsa-miR-122 (FC = −1.47), while eleven were over-expressed: hsa-miR-210 (FC = 3.79), hsa-miR-192 (FC = 1.80), hsa-miR-98 (FC = 1.71), hsa-miR-130b (FC = 1.65), hsa-miR-92a (FC = 1.52), hsa-miR-503 (FC = 1.50), hsa-let-7c (FC = 1.49), hsa-miR-31–3p (FC = 1.47), hsa-miR-22 (FC = 1.37) hsa-miR-542-3p (FC = 1.34), and hsa-miR-29a (FC = 1.28) (Supplementary Table S2).

We ran multivariable logistic regression to further examine the relationship between EV-miRNAs in follicular fluid and fertilization status. The SVA package based on our model produced five surrogate variables that accounted for any unmeasured variation within our dataset. Our full model examined 126 samples and adjusted for patient’s age, body mass index (BMI), smoking status, fertilization method, total motile sperm count, batch effects, and the aforementioned five surrogate SVA variables. In this analysis, nine of the 192 EV-miRNAs (hsa-let-7a, hsa-miR-10b-3p, hsa-miR-26b, hsa-miR-30d, hsa-miR-92a, hsa-miR-130b, hsa-miR-181a, hsa-miR-652 and hsa-miR-1290) were associated with fertilization status with a p-value of <0.05. Two of the EV-miRNAs that were found as top hits in the unadjusted fold-change analysis were also identified in the adjusted regression analysis: hsa-miR-92a (OR = 2.51, CI = [1,11, 5.67]) and hsa-miR-130b (OR = 2.07, CI = [1.10, 3.90]) (Table 2). These two EV-miRNAs were found in all or almost all the follicular fluid samples (100% and 95%, respectively) as shown in Supplementary Table S1.

**Table 2:** Overlapping results of regression and fold-change analyses for fertilization status and day-3 embryo quality.

	EV-miRNA Name	Odds Ratio^a^	Confidence Interval		Fold Change^c^	p-value^b^
Fertilization Status	hsa-miR-92a^d^	2.51	1.11	5.67	1.52	0.03
	hsa-miR-130b^d^	2.07	1.10	3.90	1.65	0.05
Day 3 Embryo Quality	hsa-miR-214^d^	0.74	0.59	0.94	−3.04	0.01
	hsa-miR-454^d^	0.81	0.66	1.00	−2.43	0.03
	hsa-miR-888^d^	1.90	1.06	3.39	2.08	0.05

^a^Models adjusted for age, BMI, smoking, batch, total motility, fertilization method, and SVA surrogate variables (Fertilization status: 5 surrogates, Day 3: 2 surrogates).

^b^EV-miRNAs statistically significant in both Mann-Whitney U test and Regression Analysis.

^c^Negative fold change is under-expressed in those that failed to fertilize compared to those that had normal fertilization or those that had impaired quality compared to top quality.

^d^p-values from Mann-Whitney U or Student t-tests.

### EV-miRNAs in follicular fluid and day 3 embryo quality

To examine possible associations between EV-miRNAs in follicular fluid and day 3 embryo quality, we compared cases of top quality embryos on day 3 (n = 42) to day 3 embryos with impaired quality (n = 48). In an unadjusted fold-change analyses, we found four EV-miRNAs that significantly differed between the top quality and impaired quality embryos, one which was over-expressed in the impaired quality, hsa-miR-888 (FC = 2.08) and three which were under-expressed in the impaired quality group: hsa-miR-214 (FC = −3.04), hsa-miR-145 (FC = −2.44), and hsa-miR-454 (FC = −2.43) (Supplementary Table S3).

We further examined the association between the EV-miRNAs in follicular fluid and day 3 embryo quality using mixed-effects logistic regression. We found seven EV-miRNAs were associated with day-3 embryo quality (hsa-miR-99a, hsa-miR-130b, hsa-miR-148a, hsa-miR-184, hsa-miR-214, hsa-miR-454, hsa-miR-888), three of which overlapped with the unadjusted FC analysis, hsa-miR-214 (OR = 0.74, CI = [0.59, 0.94]), hsa-miR-454 (OR = 0.81, CI = [0.66, 1.00]), and hsa-miR-888 (OR = 1.90, CI = [1.06, 3.39]) (Table 2). These EV-miRNAs (miR-214 and miR-454 and miR-888) were identified in 41%, 49% and 18%, of the FF samples, respectively **(**Supplementary Table S1).

### Pathway analyses

**Figure 2:**
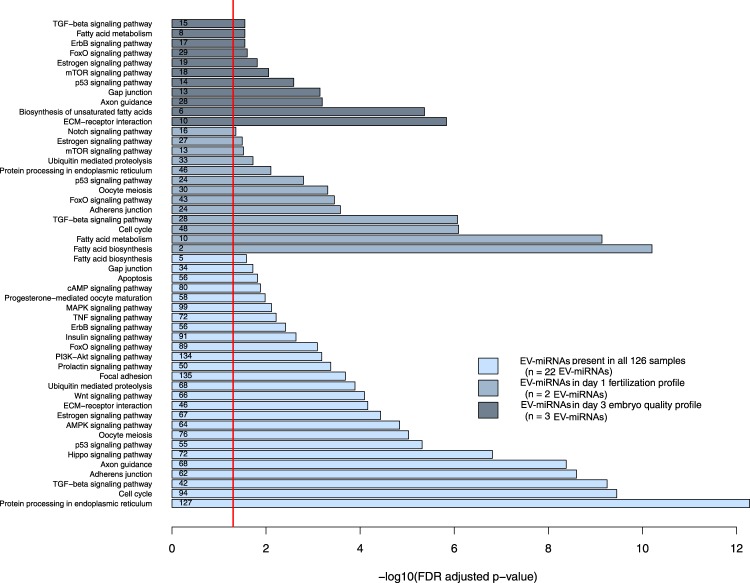
Pathways analyses – Functional pathways of EV-miRNAs using DIANA Tools miRPath-v3.0. Light blue bars represent pathways enriched among extracellular-packaged miRNAs (EV-miRNAs) detected in all follicular fluid samples, blue bars represent those pathways enriched among EV-miRNAs that were significant in the fertilization status analyses, and the dark blue bars represent pathways enriched among EV-miRNAs that were significant in the day three embryo quality analyses. The red line represents the statistical significant FDR threshold (FDR < 0.05) and the small numbers within each bar are the number of genes found to be associated with those EV-miRNAs.

Kyoto Encyclopedia of Genes and Genomes (KEGG) pathway analysis on the 22 EV-miRNAs that were 100% detectable in follicular fluid from all samples showed significant enrichment in the protein processing in endoplasmic reticulum, cell cycle, TGF-beta signaling, adherens junction, axon guidance, Hippo signaling, p53 signaling, oocyte meiosis, AMPK signaling, estrogen signaling, ECM-receptor interaction, Wnt signaling, ubiquitin mediated proteolysis, focal adhesion, prolactin signaling, PI3K-Akt signaling, FoxO, Insulin signaling, ErbB signaling, TNF signaling, MAPK signaling, progesterone-mediate oocyte maturation, cAMP signaling, apoptosis, gap junction, and fatty acid biosynthesis pathways (Fig. 2).

We also ran pathway analyses examining the two EV-miRNAs that appeared as significant in both the FC and regression analyses for fertilization status (hsa-miR-92a and hsa-miR-130b). We found several pathways that involve at least one of the EV-miRNAs including, fatty acid biosynthesis, fatty acid metabolism, cell cycle, TGF-beta signaling, adherens junction, FoxO signaling, oocyte meiosis, p53 signaling pathway, protein processing in endoplasmic reticulum, ubiquitin mediate proteolysis, mTOR signaling, estrogen signaling, and notch signaling pathways. (Fig. 2).

Last, we examined the three EV-miRNAs (hsa-miR-888, hsa-miR-454, hsa-miR-214) that were identified the FC and regression analyses in day three embryo quality EV-miRNA. Significant pathways for these three EV-miRNAs included ECM-receptor interaction, biosynthesis of unsaturated fatty acids, axon guidance, gap junction, p53 signaling, mTOR signaling, estrogen signaling, FoxO signaling, ErbB signaling, fatty acid metabolism and TGF-beta signaling pathways (Fig. 2).

## Discussion

In this study, investigating any association between EV-miRNA profiles in follicular fluid and oocyte quality, we identified two top hit EV-miRNAs (hsa-miR-130b and hsa-miR-92a) that were different among those follicles that contained oocytes that were normally fertilized compared with those that failed to fertilize. Additionally, we detected three top hit EV-miRNAs (hsa-miR-214, hsa-miR-454, and hsa-miR-888) that were different among follicular fluid that contained oocytes that further developed into embryos that were categorized as top quality at day three compared to those that were not top quality in both types of analyses.

We identified 22 EV-miRNAs that were detected in all our follicular fluid samples, including hsa-miR-16, hsa-miR-17, hsa-miR20a, hsa-miR-26a, hsa-miR-28, hsa-miR-30a-5p, hsa-miR-30b, hsa-miR-92a, hsa-miR-99b-3p, hsa-miR106a, hsa-miR-106b, hsa-miR132, hsa-miR-193b-5p, hsa-miR-203, hsa-miR-212, hsa-miR-218, hsa-miR-223, hsa-miR-320, hsa-miR-328, hsa-miR-331, hsa-miR-483-5p, and hsa-miR-1274B. Many of these EV-miRNAs were also reported earlier studies, either in mares^7^, bovine^6,22^ or human including our previous exploratory study^20,21,23^. This suggests that EV-miRNAs in follicular fluid might be conserved across species.

Pathway analyses of these EV-miRNAs that were detected in all samples of follicular fluid in our study showed that they were associated with growth, development, and signaling pathways, including TGF-beta, MAPK, ErbB, Wnt, PI3K-Akt and Hippo pathways. These pathways are responsible for growth and maintenance of organ size^24^, follicular development^25,26^ and oocyte maturation, further suggesting that these EV-miRNAs may play a role in oogenesis^27–31^, modulate granulosa and cumulus cell function or the oviductal epithelia, since the follicular fluid is in contact with the oviduct after ovulation.

While recent studies investigating EV-miRNAs in follicular fluid have found associations among women diagnosed with PCOS (polycystic ovary syndrome), oocyte maturation, and age^18,23,32–37^, few studies to date have investigated possible associations between EV-miRNAs and IVF outcomes as fertilization status and embryo quality on day three^21,38,39^. Normal fertilization is a pre-requisite for embryo development and high-quality embryos on day 3 result in higher chances for successful pregnancy.

In the current analysis, we identified hsa-miR-130b and hsa-miR-92a that were significantly different among those follicles that contained oocytes that were normally fertilized compared with those that failed to fertilize. In bovine, miR-130b is involved in granulosa and cumulus cells function, oocyte maturation and blastocyst formation^40^. Both hsa-miR-130b and hsa-miR-92a were not detected in our previous analysis when we used exoRNeasy - affinity-based isolation kit for the isolation of EVs. In our previous explorative study^21^, we identified four EV-miRNAs (miR-202-5p, miR-206, miR 16-1-3p and miR-1244) with significant fold change when we compared follicular fluid that contained normally fertilized oocytes with follicular fluid that contained failed to fertilize oocytes. Of note, both miR-206 and miR-1244 were undetected in our current analysis when we isolated EVs using ultracentrifugation. Interestingly, while in the current analysis miR-10b-3p was significantly associated with fertilization status after multivariable logistic regression, miR-10b-3p was one of the top-20 EV-miRNA with fold change expression although the p value was not significant in our previous study. A previous study has shown that miR-10 repress granulosa cells development during folliculogenesis and hormones and growth factors in the follicle, such as FSH, FGF9 and ligands in the TGF-β pathway, inhibit miR-10b expression in granulosa cells^41^. Moreover, miR-20 was also among the top 20 EV-miRNAs for fertilization in both studies, but the change in its expression was not statistically significant. As of the large biological variability, it might give a hint that these EV-miRNAs may have a role in fertilization.

We identified three EV-miRNAs (hsa-miR-214, hsa-miR-454, and hsa-miR-888) that were differently expressed in the follicular fluid  that yielded day 3 top quality embryos (containing 7–8 cells and less than 10% fragments versus non-top quality day 3 embryos. In our previous analysis, hsa-miR-454 was differently expressed in FF that contained normal fertilized oocytes vs. abnormally fertilized oocytes (p < 0.0001) and also was one of the top EV-miRNA that their fold change expression differed between follicular fluid that contained top and non-top quality day 3 embryos (p values non-significant).

The results from the current study did not overlap with our previous study in which we identified miR-766-3p, miR-663b, miR-132-3p and miR-16-5p that were differentially expressed in the follicular fluid that yielded day 3 top quality embryos versus non-top quality day 3 embryos^21^. As we used two different approaches to isolate EV-miRNAs, it seems that the two studies are not comparable. Both miR-214 and miR-888 were not detected in the previous study and miR-663 and miR-16-5p were not isolated in the current study. Also the detection rates of miR-766 and miR-132 differed for both studies which might attribute to the dissimilar results (Supplementary Table S4). Another human study looked at miRNA profile and day-3 embryo quality. They defined embryos as high quality (containing seven or more cells) or low quality (containing six or fewer cells), and examined 15 candidate miRNAs (miR-222, miR-320, miR-24, miR-132, let-7b, miR-106a, miR-19b, miR-16, miR-186, miR-339-3p, miR-17, miR-323-3p, miR-197, miR-20a, and miR-382). They found two miRNAs (hsa-miR-320 and hsa-miR-197) were down-regulated in those embryos with low quality compared to those embryos of high quality^38^. While our results were not significant, we also found that hsa-miR-320 was also down-regulated in the follicular fluid of those oocytes ultimately associated with impaired embryo quality. Another study examining EV-miRNAs in bovine samples *in vitro* found expression of miR-24 to be higher in the media retrieved from degenerate embryos (those that failed to develop the morula to blastocyst stage) compared with blastocyst media. We found hsa-miR-24 was expressed, but not significant, in the opposite direction as its bovine counterpart, which could be due to differences in tissues examined^39^.

We further investigated the targets of the EV-miRNAs that were different between follicles that contained fertilized vs. non-fertilized oocytes or day 3 embryos with top or impaired quality. Of these EV-miRNAs, hsa-miR-130b modulates oocyte competence by targeting SMAD5 and MSK1 genes^40^ and both hsa-miR-92a and hsa-miR-214 belong to the cluster 17–92, that targets the PTEN-PI3K-Akt pathway and controls oocyte or follicular development^42,43^. In addition, these EV-miRNAs are linked to pathways of interest for IVF-outcomes including cellular signaling, follicular growth, oocyte meiosis, and follicular development as TGF-Beta signaling, FoxO signaling, Estrogen signaling, mTOR signaling, and fatty acid metabolism^15,44–46^.

Our study has several limitations. While we found significance with several EV-miRNAs, after accounting for multiple testing, our associations did not remain significant using FDR adjustments. Our results might be subjected to spurious associations or false-positives. We suppose this is due to the highly correlated nature of EV-miRNAs and high biological variability between samples and that in this case, we rather not perform correction for multiple comparisons, in order to prioritize the biological relevance of the data than a statistical correction. However, this can be overcome using a larger sample size, but this is something we could not control for. We believe that the overlap of marginal significant EV-miRNAs (p < 0.05) in two separate statistical analyses (unadjusted analysis using Mann-Whitney U or Student *t-*tests and calculated fold-change (FC) differences and multivariable logistic regression may provide insight of biological relevance. These observed results only allow for evaluation of associations and further investigation is necessary to understand further, the biological mechanisms that these EV-miRNAs might play. Despite this, and as this study is the largest study to date looking at EV-miRNAs in human follicular fluid, we believe that those EV-miRNAs that were significant in the unadjusted models and remained significant after adjusting for confounders are more robust and could provide biological relevance. Moreover, most previous studies did not account for multiple testing, suggesting that the biological variability between patients is too high to find any FDR corrected significance and that a much larger sample size is needed. Using follicular fluid we were unable to distinguish whether these EV-miRNAs originate from the cumulus cells, granulosa cells, or from the oocyte itself and there is a possibility that EV-miRNAs in follicular fluid might not be a useable biomarker for these outcomes. Even so, we have identified top hit EV-miRNAs that were expressed differently in fertilization status and day three embryo quality. Additionally, our pathway analyses were run on a very small number of EV-miRNAs, so it is hard to determine whether there was over fitting of the pathways selected by the software. Also, all the patients in our study, similar to previous studies in the field, underwent ovarian stimulation, which might affect their EV-miRNA profile^47^. In order to minimize alterations in EV-miRNAs associated with ovarian stimulation, we enrolled only women treated with GnRH antagonists. In addition, the results of the current study did not replicate the results from our previous study. As the field of extracellular vesicles is evolving, in our current study we used different laboratory methods for isolating the EV-miRNAs (ultra-centrifugation vs. exoRNeasy - affinity-based isolation kit). In the past years, ultra-centrifugation has become the gold standard for the isolation of extracellular vesicles according to the International Society of Extracellular Vesicles. The affinity-based methods can yield pure EV subpopulations, but those subpopulations are highly dependent on the affinity reagent and ligand density, which can alter which EVs will be captured^48–50^. Therefore, it is possible that some of the differences are attributed to different composition of various types of extracellular vesicles. Moreover, between the first and this current study, we gained extensive experience through a large study we conducted on 2000 subjects and the choice of a more strict Ct threshold (28 vs 35) was supported by these new methods^51,52^. Additionally, we did not consider the frequency of detection (expression) of the EV-miRNAs. It is unclear whether it is more accurate to choose as biomarkers EV-miRNAs that are more prevalent but their expression varies or those that are present only in small percentages of the samples. Further studies are needed to compare EV-miRNA profile according to these IVF outcomes. Another important question that could not be answered in the current study was whether EV-miRNAs can predict pregnancies. In the current study, we followed one single follicle from each IVF cohort. As usually we retrieve many follicles for each case, the chances that the embryo that will be transferred will derive from the single follicle we have included in the study are not so high (5–10%). To address the question, whether EV-miRNAs from follicular fluid associated with the prediction of pregnancies, we should have collected all follicles separately and include in our analysis only cases of single embryo transfer. This was not feasible in our study population. Despite these limitations, our study has strengths. This is one of the largest studies to date that has examined EV-miRNAs in human follicular fluid and it is the largest study examining EV-miRNAs in follicular fluid with fertilization status and embryo quality. We were also able to account for potential confounders by examining EV-miRNAs using logistic regression, allowing us to start to tease apart a true association.

This study expands the growing understanding of the human follicular fluid profile and how EV-miRNAs may play a role in inter-cellular communication among the mural and cumulus granulosa cells and the oocyte. We caution that our findings might be subject to false-positives. However, we believe that these findings are novel and that they could provide a biological basis for future research. Larger studies with specific ad-hoc analysis on specific populations are warranted. Understanding the role of these EV-miRNAs and how they might interact or regulate pathways of ovarian function, follicular and oocyte development and growth will expand our understanding of the physiology of reproduction and may allow us to identify them as non-invasive biomarkers of oocyte health. Such studies should assist in the development of targeted treatments of infertility.

## Methods

### Ethics Statement

This study was approved by Sheba Medical Center IRB in accordance with the Declaration of Helsinki^53^. Authors confirm that all methods were in accordance with the relevant guidelines and regulations. All participants provided written informed consent upon enrollment.

### Participants

Women 19 to 38 years old undergoing IVF and IVF/ICSI in a tertiary care university-affiliated hospital from January 2014 through August 2016 with ≤6 previous attempts were eligible for recruitment. Exclusion criteria were a diagnosis of polycystic ovarian syndrome (PCOS), endometriosis, poor responders according to Bologna criteria^54^ and/or cycles having severe male factor infertility. To avoid potential confounding by the stimulation protocol, only women using one regimen (antagonist protocol) were included. Each woman was represented in only one IVF cycle.

### IVF Protocols and sample collection

Follicular fluid (otherwise discarded material) from one or two single follicles >18 mm was collected and centrifuged at 1500 × g for 15 min. Samples were coded, filtered using a 0.80 um pore-size polyethersulfone filter (StericupRVP, Merck Millipore), aliquoted into 500 ul, and stored immediately at −80 °C. Each oocyte, its corresponding embryo, derived from the follicle collected separately tracked in the clinical IVF laboratory. To ensure blinded samples for outcome measures, each related follicular fluid sample was coded using an anonymized ID.

In cases of ICSI, oocytes were stripped and assessed for maturity 1–2 hours after retrieval. Only follicular fluid from single follicles that contained mature oocytes (i.e., those exhibiting one polar body) were analyzed. Oocyte maturity in conventional IVF was assessed as per standard protocol after removal of the cumulus/corona radiata cells at the fertilization check. The total number of mature oocytes in a conventional IVF cycle was determined by summing the number of oocytes exhibiting one or more pronucleus combined with those without a pronucleus but exhibiting a polar body. Oocytes and embryos were incubated at 37 °C humidified atmosphere of 5% CO_2_ in air^55^. The fertilization check was performed approximately 16–18 hours after ICSI and standard insemination. Morphology of the embryo was assessed on day three, 72 hours after oocyte retrieval, using standard criteria based on the number of blastomeres, symmetry, and the extent of fragmentation^56,57^.

### EV-miRNA Exposure Assessment

#### RNA extraction from Follicular Fluid

Methods for RNA extraction from follicular fluid have been previously described elsewhere^52^; however in short, samples were thawed and centrifuged for 15 min at 1200 × g at room temperature. They were subsequently centrifuged at 1000, 2000, and 3000 × g for 15 min at 4 °C. The samples were then ultra-centrifuged for the extraction of EV, as ultracentrifugation is considered the standard according to International Society for Extracellular Vesicle recommendations^58^, (Beckman Coulter Optima-MAX-XP) at 110,000 × g for 75 min at 4 °C. The pellets obtained were kept at −80 °C until use. EV-miRNAs were extracted from the aforementioned follicular fluid pellets using the miRNAeasyKit and RNeasy CleanUp Kit as described (Qiagen, Valencia, CA, USA). The final purified miRNA-enriched RNA was eluted into 20 ul of RNAse-free water and stored at −80 °C until further use.

#### Expression analysis of EV miRNAs in follicular fluid

We screened for levels of 754 EV-miRNAs using the TaqMan Open Array® system. We obtained 758 Crt values for each follicular fluid sample, which included 754 unique miRNAs and four internal controls (ath-miR159a, RNU48, RNU44 and U6). Methods of Real-Time Quantitative Polymerase Chain Reaction (RT-qPCR) for screening EV-miRNAs in follicular fluid are found elsewhere^52^. QuantStudio™ 12 K Flex is a fixed-content panel containing validated human TaqMan® MicroRNA Assays derived from Sanger miRBase release v.14. All 754 assays have been functionally validated with miRNA artificial templates. The panel is specifically designed to provide specificity for only the mature miRNA targets. TaqMan MicroRNA Assays (spotted in the panel) incorporate a target-specific stem–loop reverse transcription primer allowing to work despite the short length of mature miRNAs (~22 nucleotides) which prohibits conventional design of primers. In short, reverse transcription was performed by using Megaplex™ reverse transcription Primers, Pool A v2.1 and Pool B v3.0, with the TaqMan® Micro RNA Reverse Transcriptase Kit (Life Technologies, Foster City, CA). Two distinct reactions were performed, to cover reverse transcription of the 754 target miRNAs (16 replicates of four internal controls: ath-miR159a, RNU48, RNU44, and U6). Each reaction included: 0.75 µl of Megaplex RT Primers Pool A or Pool B, 0.15 µl of dNTPs (100 mM), 0.75 µl of 10× RT Buffer, 0.90 µl of MgCl2 (25 mM), 0.1 µl of RNase Inhibitor (20 U/µl), 1.5 µl of MultiScribe™ Reverse Transcriptase (50 U/µl), and 3.3 µl of EV-miRNAs. Negative controls were run to ensure quality of the assay. After incubation on ice for 5 min, the mixture was subjected to the following thermal protocol in a C1000 Thermal Cycler (Biorad, Hercules, CA): 40 cycles of 16 °C for 2 min, 42 °C for 1 min, and 50 °C for 1 s, plus one cycle at 85 °C for 5 min. The cDNA (complementary DNA) samples were stored at −20 °C until use.

Each cDNA requiring preamplification was loaded onto a 96-well plate in accordance with the protocol provided by the manufacturer (Application Note 2011, Life Technologies). 7.5 μl of each reverse-transcribed EV-miRNA was combined with the following reaction mix: 20 µl of TaqMan® PreAmp Master Mix (2×), 8.5 µl of nuclease-free water, and 4 µl of Megaplex™ PreAmp Primers Pool A or B (10×). Thermal conditions for the preamplification reaction were as follows: 95 °C for 10 min, 55 °C for 2 min, 72 °C for 2 min, 16 cycles of 95 °C for 15 s and 60 °C for 4 min, and 99.9 °C for 10 min. Preamplified samples were stored at 4 °C until expression analysis with the OpenArray® System. Each preamplified cDNA was diluted 1:20 with nuclease-free water. TaqMan Open Array® Real Time PCR Master Mix (2×) was added in a 1:1 volume ratio. Then, 7 µl of the reaction RT-PCR mix was aliquoted with the MicroLab STAR Let instrument (Hamilton Robotics, Birmingham, UK) into eight wells of a 384-well OpenArray® plate. The reaction mix was loaded from the 384-well plate into a TaqMan™ OpenArray® Human miRNA Panel, with QuantStudio™ AccuFill System Robot (Life Technologies, Foster City, CA). RT-qPCR was performed on the QuantStudio™ 12 K Flex Real-Time PCR System with the OpenArray® Platform [QS12KFLEX] (Life Technologies, Carlsbad, CA) according to the manufacturer’s instructions. Expression levels were calculated in relative cycle threshold values (Crt), which estimate the amplification cycle at which the fluorescence levels for each of the analyzed miRNAs exceed the background fluorescence threshold^59^.

### Covariate Assessment

Patients’ age, BMI (body mass index), fertility diagnosis (male factor, PGD [preimplantation genetic diagnosis], unexplained, mechanical, sexual dysfunction, and egg donor), smoking status, number of previous IVF attempts, number of oocytes retrieved at the index of IVF cycle, method of fertilization (ICSI [intracytoplasmic sperm injection] or insemination), and total motile sperm count were obtained from the medical charts. We also accounted for when the follicular fluid samples were sent from Israel to Milan for EV-miRNA analysis (batch 1 vs batch 2). As all follicular fluid samples were coded, they were run blindly and the laboratory in Milan did not have access to any of the participants’ data, including the clinical outcomes of the oocyte embedded in the follicle (i.e., fertilization status and day 3 embryo quality).

### Outcome Variable Assessment

Oocytes that fertilized normally were defined as those exhibiting two pronuclei and two polar bodies on day one post-fertilization. Samples that were abnormally fertilized were excluded from our study. Embryo quality and morphology were assessed on day three using the standard criteria of the number of blastomeres, symmetry and extent of fragmentation^57,60^. Top quality embryos on day 3 were classified as those having 7–8 cells and ≤10% fragmentation. All others were classified as poor-quality embryos.

### Data analysis

We used the Thermo Fisher Cloud Relative Quantification software to extract the miRNA qPCR data. We normalized our EV-miRNA data using the global mean (GM) method (∆C_rt_EV-miRNAi_ =(C_rt_EV-miRNAi_ − C_rt_EV-miRNAi_global_mean_)) as suggested by Pergoli *et al*.^52^. First, we considered as unexpressed all the EV-miRNA with a Crt value >28 and/or an amplification score ≤1.24. For the global mean, we coded all those EV-miRNAs that were unexpressed as 28. We calculated the delta Crt based on the global mean across all the miRNAs within that subject and dividing it by the total miRNAs (N = 754). Standard descriptive statistics were used to explore the characteristics of the study participants. To examine fold change, we calculated the relative expression levels between two groups (either fertilized and non-fertilized oocytes and top embryo vs. non top embryo on day 3) using the 2^−∆∆Crt^ formula^61^. Relative expression between groups were compared using Student t-tests and Mann Whitney U tests for statistical significance as appropriate based on the Shapiro-Wilk’s test for normality^62^. Adjusted logistic regression models with were performed to further elucidate top hit EV-miRNAs. Models were all adjusted for *a priori* covariates: age, BMI, smoking status, method of fertilization, total motile sperm count, and batch number. Regression analyses were further adjusted for any unwanted variation within our high-throughput assay by applying the SVA package^63^. The SVA package can help identify and remove any batch effects or unwanted sources of variation, seasonal, meteorological, exposure, or technical variables, which are unknown but might be differently distributed in the two batches of samples. It creates surrogate variables, accounting for the unmeasured variation, that act as covariates in our models that would account for any unknown, un-modeled, or other sources of noise^64^. All EV-miRNAs for regression analyses were inverse-normally transformed to ensure a Gaussian distribution. All statistical analyses were performed in R-version 3.4.0^65^. Statistical significance was set at a p-value ≤ 0.05.

### Target Gene Predictions and Pathway Analysis

We performed an *in-silico* analysis using a web-based tool DIANA miRPath-v3.0 (http://www.microrna.gr/miRPathv3) to investigate pathways on EV-miRNAs that were identified in both the fold-change and regression analyses^66^. We analyzed both the *in-silico* predictions (DIANA-microT-CDS v5.0) and the experimentally validated EV-miRNA-gene interactions (DIANA TarBase v7.0). DIANA-microT-CDS adopts an algorithm which detects and scores both the EV-miRNA binding sites in the 3′-UTR and coding sequences of predicted target genes. We used the default threshold score of 0.8^66^. The DIANA TarBase v7.0 examined the union of genes and pathways targeted by at least one of the selected EV-miRNAs and performs a Fisher’s combined probability method to calculated a merged p-value. Significant results for pathway analyses are adjusted for FDR (p < 0.05).

## Electronic supplementary material

Supplementary Materials

## Data Availability

The datasets analyzed during the current study are not publicly available due to protection of participant confidentiality but are available from the corresponding author on reasonable request with assurances and plans in place to protect confidentiality.
