# Supplementary material for: Extracellular microRNAs profile in human follicular fluid and IVF outcomes
Source: Sci Rep. 2018 Nov 19;8:17036. doi: 10.1038/s41598-018-35379-3 (PMC6242846; doi:10.1038/s41598-018-35379-3)
Supplement: Supplementary file 1 — Supplementary Materials [file 41598_2018_35379_MOESM1_ESM.pdf]

Extracellular microRNAs profile in human follicular fluid and IVF outcomes

Rosie M Martinez[1], Liming Liang[2], Catherine Racowsky[3], Laura Dioni[4], Abdallah Mansur[5], Michal Adir[5], Valentina Bollati[4], Andrea A Baccarelli[6], Russ Hauser[7], Ronit Machtinger [5,*]

Supplemental Table S1: All EV-miRNAs and their % detected, mean Ct and SD of normalized and unnormalized data among 126 samples					
Mature EV-miRNA name	Percent Detected in 126 FF Samples	Mean Ct of Normalized Data ^	SD Ct of Normalized Data	Mean Ct of Unnormalized Data **	SD Ct of Unnormalized Data
hsa-miR-106a-5p	100%	-8.22	1.08	18.84	1.43
hsa-miR-106b-5p	100%	-5.50	1.12	21.56	1.47
hsa-miR-1274B	100%	-12.14	1.12	14.93	1.42
hsa-miR-132-3p	100%	-10.25	1.59	16.82	1.91
hsa-miR-16-5p	100%	-5.27	1.61	21.79	1.95
hsa-miR-17-5p	100%	-8.32	1.06	18.75	1.41
hsa-miR-193b-3p	100%	-9.76	1.21	17.30	1.50
hsa-miR-203a-3p	100%	-6.15	1.25	20.91	1.52
hsa-miR-20a-5p	100%	-9.81	1.08	17.25	1.42
hsa-miR-212-3p	100%	-6.51	1.27	20.55	1.57
hsa-miR-218-5p	100%	-4.05	1.18	23.01	1.50
hsa-miR-223-3p	100%	-8.20	2.04	18.86	2.29
hsa-miR-26a-5p	100%	-6.10	1.32	20.96	1.66
hsa-miR-28-5p	100%	-4.45	1.24	22.62	1.58
hsa-miR-30a-5p	100%	-7.43	1.45	19.63	1.79
hsa-miR-30b-5p	100%	-9.20	1.36	17.86	1.69
hsa-miR-320a	100%	-8.74	1.27	18.32	1.59
hsa-miR-328-3p	100%	-6.78	1.31	20.28	1.64
hsa-miR-331-3p	100%	-5.84	1.16	21.22	1.50
hsa-miR-483-5p	100%	-10.31	1.25	16.75	1.49
hsa-miR-92a-3p	100%	-9.50	1.27	17.56	1.60
hsa-miR-99b-5p	100%	-7.25	1.20	19.82	1.55
hsa-let-7c-5p	99%	-4.96	1.42	22.10	1.75
hsa-miR-127-3p	99%	-5.96	1.33	21.10	1.61
hsa-miR-130a-3p	99%	-5.01	1.69	22.05	2.04
hsa-miR-146b-5p	99%	-3.86	1.57	23.20	1.91
hsa-miR-191-5p	99%	-7.88	1.41	19.18	1.72
hsa-miR-195-5p	99%	-4.39	1.51	22.67	1.86
hsa-miR-26b-5p	99%	-4.44	1.36	22.62	1.69
hsa-miR-720	99%	-7.74	1.22	19.32	1.48
hsa-miR-886-3p	99%	-4.99	1.67	22.07	1.90
hsa-miR-125b-5p	98%	-7.23	1.82	19.83	2.13
hsa-miR-15b-5p	98%	-4.90	1.36	22.16	1.70
hsa-miR-19b-3p	98%	-9.23	1.92	17.83	2.17
hsa-miR-202-3p	98%	-4.50	1.60	22.56	1.87
hsa-miR-24-3p	98%	-8.32	1.76	18.74	2.04
hsa-miR-30c-5p	98%	-9.23	1.86	17.83	2.15
hsa-miR-451a	98%	-5.85	2.22	21.22	2.44

hsa-miR-25-3p	98%	-4.66	1.72	22.40	2.06
hsa-miR-30d-5p	98%	-3.97	1.69	23.09	2.02
hsa-miR-374a-5p	98%	-3.37	1.46	23.69	1.78
hsa-miR-484	98%	-6.85	1.81	20.21	2.10
hsa-miR-572	98%	-2.34	1.59	24.72	1.61
hsa-miR-1274A	97%	-6.66	1.79	20.40	2.06
hsa-miR-150-5p	97%	-4.99	1.87	22.07	2.15
hsa-miR-130b-3p	95%	-3.02	1.50	24.04	1.83
hsa-miR-29a-3p	95%	-3.08	1.67	23.98	2.02
hsa-miR-31-5	95%	-4.74	1.62	22.32	1.91
hsa-miR-376a-3p	95%	-3.44	1.48	23.62	1.79
hsa-miR-424-3p	95%	-4.73	1.56	22.33	1.79
hsa-miR-590-5p	95%	-2.60	1.49	24.46	1.78
hsa-miR-103a-3p	94%	-3.22	1.45	23.84	1.76
hsa-miR-146a-5p	94%	-3.93	2.05	23.13	2.37
hsa-miR-324-5p	94%	-2.79	1.39	24.27	1.71
hsa-miR-574-3p	94%	-6.10	1.93	20.96	2.23
hsa-miR-424-5p	94%	-3.14	1.55	23.92	1.88
hsa-miR-503-5p	94%	-2.54	1.23	24.53	1.53
hsa-miR-99a-5p	94%	-5.66	2.00	21.40	2.30
hsa-miR-335-5p	93%	-2.94	1.68	24.12	1.99
hsa-miR-192-5p	92%	-2.89	1.78	24.18	1.98
hsa-miR-19a-3p	91%	-3.38	1.70	23.68	2.02
hsa-miR-375	91%	-3.17	1.79	23.89	2.10
hsa-miR-152-3p	90%	-3.09	1.71	23.97	2.04
hsa-miR-411-5p	90%	-2.31	1.41	24.76	1.71
hsa-miR-27b-3p	90%	-3.16	1.59	23.90	1.91
hsa-miR-660-5p	90%	-2.66	1.59	24.40	1.93
hsa-miR-126-3p	89%	-3.11	2.16	23.96	2.46
hsa-miR-142-3p	89%	-2.92	2.05	24.14	2.33
hsa-miR-181a-5p	89%	-2.69	1.57	24.37	1.89
hsa-miR-30a-3p	89%	-4.28	2.05	22.78	2.34
hsa-miR-30e-3p	89%	-4.13	2.23	22.93	2.49
hsa-miR-374b-5p	89%	-2.38	1.47	24.68	1.79
hsa-miR-186-5p	88%	-2.44	1.70	24.62	2.02
hsa-miR-222-3p	88%	-3.35	2.16	23.71	2.48
hsa-miR-29c-3p	88%	-2.88	2.00	24.19	2.31
hsa-miR-204-5p	87%	-3.29	1.99	23.77	2.29
hsa-miR-21-5p	87%	-6.98	3.35	20.08	3.63
hsa-miR-376c-3p	87%	-2.96	1.97	24.10	2.27
hsa-miR-886-5p	87%	-2.06	1.60	25.00	1.82
hsa-miR-140-5p	86%	-2.13	1.49	24.94	1.83
hsa-miR-532-5p	85%	-2.34	1.58	24.72	1.92

hsa-miR-942-5p	85%	-1.55	1.26	25.51	1.53
hsa-let-7d-5p	83%	-1.68	1.32	25.38	1.65
hsa-miR-100-5p	82%	-4.95	2.85	22.11	3.13
hsa-let-7g-5p	81%	-1.99	1.55	25.07	1.81
hsa-miR-148a-3p	79%	-3.38	2.33	23.68	2.64
hsa-miR-301a-3p	79%	-1.51	1.36	25.55	1.68
hsa-miR-361-5p	79%	-2.19	1.68	24.87	2.01
hsa-miR-409-3p	79%	-4.31	2.70	22.75	2.93
hsa-miR-126-5p	78%	-1.75	1.86	25.31	2.15
hsa-miR-1291	78%	-1.36	1.34	25.71	1.60
hsa-miR-31-3	78%	-1.35	1.32	25.71	1.64
hsa-miR-410-3p	78%	-1.47	1.43	25.59	1.73
hsa-miR-645	78%	-0.58	1.02	26.48	0.94
hsa-miR-320b	77%	-1.37	1.40	25.69	1.70
hsa-miR-365a-3p	77%	-2.23	2.04	24.83	2.38
hsa-miR-28-3p	75%	-3.81	2.63	23.26	2.91
hsa-miR-34a-5p	75%	-4.48	2.93	22.58	3.17
hsa-miR-143-3p	75%	-1.55	1.68	25.51	1.98
hsa-miR-139-5p	73%	-1.92	1.79	25.14	2.08
hsa-miR-324-3p	73%	-1.15	1.35	25.92	1.65
hsa-miR-63	72%	-1.65	1.68	25.41	1.76
hsa-miR-93-5p	72%	-2.62	2.07	24.44	2.39
hsa-miR-423-5p	71%	-1.85	1.82	25.21	2.13
hsa-miR-509-5p	71%	-2.01	1.93	25.05	2.20
hsa-miR-210-3p	69%	-5.91	4.42	21.15	4.69
hsa-miR-221-3p	69%	-3.07	2.68	23.99	2.99
hsa-miR-296-5p	66%	-2.46	2.62	24.60	2.83
hsa-miR-29b-3p	65%	-0.96	1.38	26.10	1.68
hsa-miR-145-5p	64%	-2.33	2.67	24.73	2.93
hsa-miR-597-5p	62%	-0.55	1.16	26.51	1.24
hsa-miR-15a-5p	61%	-0.49	1.08	26.57	1.38
hsa-let-7e-5p	60%	-3.70	3.62	23.36	3.88
hsa-miR-34a-3	60%	-0.61	1.25	26.45	1.54
hsa-miR-708-5p	60%	-0.71	1.56	26.35	1.80
hsa-miR-744-5p	60%	-0.68	1.27	26.38	1.59
hsa-miR-134-5p	59%	-0.84	1.46	26.22	1.73
hsa-miR-27a-3p	58%	-1.12	1.75	25.94	2.04
hsa-miR-345-5p	58%	-0.45	1.14	26.61	1.46
hsa-miR-483-3	57%	-2.67	3.13	24.39	3.25
hsa-miR-508-3	57%	-0.69	1.41	26.37	1.69
hsa-miR-193a-5p	56%	-0.54	1.37	26.52	1.68
hsa-miR-95-3p	56%	-0.50	1.24	26.56	1.52
hsa-miR-205-5p	55%	-0.66	1.72	26.40	1.86

hsa-miR-532-3p	55%	-0.49	1.30	26.57	1.62
hsa-miR-629-3	55%	-0.37	1.16	26.69	1.40
hsa-miR-184	54%	-0.52	1.40	26.54	1.51
hsa-miR-193b-5p	54%	-1.57	2.21	25.49	2.47
hsa-miR-128-3p	53%	-0.29	1.02	26.77	1.34
hsa-miR-125a-5p	52%	-0.57	1.38	26.50	1.68
hsa-miR-10a-5p	52%	-0.60	1.80	26.46	2.02
hsa-miR-885-5p	52%	-0.78	1.96	26.28	2.22
hsa-miR-10b-3	51%	-0.68	1.64	26.38	1.93
hsa-miR-1290	50%	-0.61	1.62	26.45	1.75
hsa-miR-151a-3p	49%	-0.40	1.28	26.66	1.59
hsa-miR-22-5p	49%	-0.40	1.18	26.66	1.46
hsa-miR-454-3p	49%	-0.99	2.70	26.07	2.84
hsa-miR-543	48%	-0.03	0.91	27.03	1.15
hsa-miR-601	48%	-0.48	2.21	26.58	2.29
hsa-miR-323a-3p	46%	-0.34	1.36	26.73	1.63
hsa-miR-379-5p	46%	-0.38	1.32	26.68	1.55
hsa-miR-495-3p	45%	-0.83	1.82	26.23	2.11
hsa-miR-148b-3p	44%	-0.21	1.15	26.85	1.47
hsa-miR-135a-5p	42%	-0.26	1.49	26.81	1.72
hsa-miR-20b-5p	42%	-0.29	1.32	26.77	1.60
hsa-miR-214-3p	41%	-1.51	2.87	25.55	3.11
hsa-miR-542-3p	40%	0.11	0.92	27.17	1.14
hsa-miR-200b-3p	39%	-0.41	1.69	26.65	1.91
hsa-miR-149-5p	37%	-1.04	2.34	26.02	2.63
hsa-miR-194-5p	37%	-0.09	1.41	26.97	1.62
hsa-miR-491-5p	37%	-0.03	1.09	27.03	1.34
hsa-miR-7-1-3p	37%	-1.11	2.48	25.95	2.74
hsa-miR-29a-5p	36%	0.14	0.96	27.20	1.20
hsa-miR-497-5p	36%	0.08	1.08	27.15	1.33
hsa-miR-133a-3p	34%	-0.39	1.73	26.67	1.94
hsa-miR-185-5p	33%	0.22	0.90	27.28	1.16
hsa-miR-1303	33%	0.07	1.33	27.13	1.37
hsa-miR-1180-3p	32%	0.36	0.70	27.42	0.93
hsa-miR-455-5p	30%	0.36	0.76	27.42	1.01
hsa-miR-548b-5	30%	0.32	1.02	27.39	1.13
hsa-miR-598-3p	28%	0.39	0.74	27.46	0.97
hsa-miR-101-3p	27%	0.34	0.90	27.40	1.16
hsa-let-7a-5p	26%	-1.41	3.75	25.65	3.99
hsa-miR-10b-5p	25%	0.13	1.41	27.19	1.64
hsa-miR-1226-5	25%	0.00	1.45	27.06	1.66
hsa-miR-190a-5p	25%	0.33	1.02	27.40	1.21
hsa-miR-202-5p	25%	0.24	1.12	27.30	1.30

hsa-miR-769-5	25%	0.29	0.96	27.35	1.17
hsa-miR-99b-3p	25%	0.14	1.29	27.20	1.40
hsa-miR-18a-5p	24%	0.18	1.20	27.24	1.43
hsa-miR-339-3p	23%	0.47	0.71	27.53	0.95
hsa-miR-339-5p	23%	-0.57	2.56	26.49	2.80
hsa-miR-342-3p	23%	0.10	1.52	27.16	1.72
hsa-miR-652-3p	23%	0.22	1.23	27.28	1.47
hsa-miR-141-3p	22%	-0.24	2.19	26.82	2.36
hsa-miR-539-5p	22%	0.53	0.69	27.59	0.85
hsa-miR-381-3p	21%	0.59	0.64	27.65	0.70
hsa-miR-199a-3p	20%	-0.05	1.98	27.01	2.07
hsa-miR-122-5p	19%	0.20	1.49	27.26	1.59
hsa-miR-144-5	19%	0.30	1.32	27.36	1.50
hsa-miR-34b-3p	18%	-0.02	2.10	27.04	2.29
hsa-miR-766-3p	18%	0.14	1.55	27.20	1.76
hsa-miR-888-5p	18%	0.11	2.22	27.17	2.25
hsa-miR-98-5p	18%	-0.17	2.33	26.89	2.40
hsa-miR-155-5p	17%	0.52	1.11	27.58	1.21
hsa-miR-449b-5p	17%	0.31	1.34	27.37	1.50
hsa-miR-487b-3p	17%	0.65	0.56	27.71	0.69
hsa-miR-93-3p	17%	0.58	0.65	27.64	0.86
hsa-miR-34c-5p	16%	0.40	1.21	27.46	1.40
hsa-miR-199a-5p	15%	0.60	0.76	27.66	0.90
hsa-miR-370-3p	15%	-0.03	2.14	27.03	2.32
hsa-miR-548am-5p	15%	0.70	0.58	27.76	0.59
hsa-miR-135b-5p	14%	0.62	0.70	27.68	0.87
hsa-miR-146b-3p	14%	0.70	0.47	27.76	0.63
hsa-miR-224-5	14%	0.50	0.99	27.56	1.18
hsa-miR-889-3p	14%	0.66	0.59	27.72	0.73
hsa-miR-136-3p	13%	0.71	0.51	27.77	0.63
hsa-miR-206	13%	0.70	0.72	27.76	0.71
hsa-miR-362-5p	13%	0.66	0.60	27.73	0.74
hsa-miR-502-5p	13%	0.71	0.55	27.77	0.63
hsa-miR-372-3p	13%	0.76	0.50	27.82	0.51
hsa-miR-506-3p	12%	0.68	0.67	27.74	0.77
hsa-miR-505-3p	11%	0.71	0.53	27.77	0.66
hsa-miR-625-3	11%	0.65	0.83	27.71	0.92
hsa-miR-124-3p	10%	0.75	0.54	27.81	0.59
hsa-miR-369-3p	10%	0.01	2.62	27.07	2.76
hsa-miR-382-5p	10%	0.68	0.73	27.74	0.83
hsa-miR-449a	10%	0.76	0.47	27.82	0.55
hsa-miR-488-5p	10%	0.72	0.64	27.78	0.72
hsa-miR-494-3p	10%	0.76	0.51	27.82	0.54

hsa-miR-548d-5	10%	0.36	2.09	27.42	2.13
hsa-miR-758-3p	10%	0.78	0.52	27.84	0.49
hsa-miR-1244	10%	0.80	0.46	27.86	0.45
hsa-miR-520c-3p	10%	0.59	1.30	27.65	1.30
hsa-miR-154-3p	9%	0.82	0.48	27.88	0.40
hsa-miR-425-3p	9%	0.77	0.51	27.83	0.58
hsa-miR-373-3p	8%	0.82	0.54	27.88	0.42
hsa-miR-429	8%	0.78	0.57	27.84	0.64
hsa-miR-500a-5p	8%	0.77	0.51	27.83	0.61
hsa-miR-642a-5p	8%	0.70	0.76	27.76	0.89
hsa-miR-107	7%	0.82	0.47	27.88	0.46
hsa-miR-151a-5p	7%	0.80	0.45	27.86	0.54
hsa-miR-181a-2-3	7%	0.83	0.38	27.89	0.43
hsa-miR-200c-3p	7%	0.32	2.14	27.38	2.26
hsa-miR-330-3p	7%	0.76	0.62	27.82	0.72
hsa-miR-340-3p	7%	0.82	0.43	27.88	0.46
hsa-miR-378	7%	0.77	0.57	27.83	0.65
hsa-miR-576-3p	7%	0.82	0.42	27.88	0.44
hsa-miR-663b	7%	0.63	1.19	27.69	1.18
hsa-miR-744-3p	7%	0.83	0.45	27.89	0.42
hsa-miR-99a-3	7%	0.82	0.41	27.88	0.46
hsa-miR-1271-5p	6%	0.82	0.45	27.88	0.48
hsa-miR-383-5p	6%	0.56	1.31	27.62	1.47
hsa-miR-432-5p	6%	0.82	0.52	27.88	0.48
hsa-miR-510-5	6%	0.78	0.67	27.84	0.64
hsa-miR-1227-3	6%	0.82	0.56	27.88	0.51
hsa-miR-140-3p	6%	0.85	0.37	27.91	0.39
hsa-miR-20a-3	6%	0.84	0.40	27.91	0.41
hsa-miR-223-5	6%	0.85	0.41	27.91	0.38
hsa-miR-27a-5p	6%	0.85	0.37	27.91	0.36
hsa-miR-27b-5p	6%	0.85	0.40	27.91	0.39
hsa-miR-520b	6%	0.87	0.47	27.93	0.29
hsa-miR-520e	6%	0.62	1.67	27.69	1.67
hsa-miR-590-3p	6%	0.85	0.39	27.91	0.40
hsa-miR-9-5p	6%	0.67	1.06	27.73	1.16
hsa-miR-200a-3p	5%	0.85	0.46	27.91	0.44
hsa-miR-337-5p	5%	0.53	1.78	27.59	1.87
hsa-miR-338-5P	5%	0.88	0.39	27.94	0.28
hsa-miR-34b-5	5%	0.83	0.70	27.89	0.54
hsa-miR-380-5	5%	0.77	0.70	27.83	0.78
hsa-miR-450a-5p	5%	0.86	0.45	27.92	0.41
hsa-miR-622	5%	0.87	0.40	27.93	0.31
hsa-miR-7-5p	5%	0.88	0.42	27.94	0.25

hsa-let-7f-5p	4%	0.68	1.29	27.74	1.30
hsa-miR-125b-1-3p	4%	0.85	0.51	27.91	0.47
hsa-miR-197-3p	4%	0.66	1.40	27.72	1.38
hsa-miR-199b-5p	4%	0.85	0.55	27.91	0.54
hsa-miR-340-5p	4%	0.88	0.37	27.94	0.28
hsa-miR-452-5p	4%	0.85	0.47	27.91	0.48
hsa-miR-502-3p	4%	0.86	0.41	27.92	0.41
hsa-miR-659-3	4%	0.78	0.88	27.84	0.80
hsa-miR-125b-2-3	3%	0.80	0.73	27.86	0.79
hsa-miR-181c-5p	3%	0.90	0.36	27.96	0.24
hsa-miR-190b	3%	0.89	0.39	27.95	0.30
hsa-miR-302a-3p	3%	0.61	1.93	27.67	1.90
hsa-miR-30d-3p	3%	0.90	0.37	27.96	0.21
hsa-miR-329-3p	3%	0.88	0.42	27.95	0.31
hsa-miR-605-5p	3%	0.89	0.45	27.95	0.30
hsa-miR-624-5	3%	0.89	0.40	27.95	0.28
hsa-miR-63	3%	0.76	1.12	27.82	1.07
hsa-miR-671-3p	3%	0.89	0.42	27.95	0.31
hsa-let-7b-5p	2%	0.78	1.11	27.84	1.05
hsa-miR-16-1-3p	2%	0.91	0.42	27.97	0.19
hsa-miR-222-5	2%	0.87	0.49	27.93	0.47
hsa-miR-301b-3p	2%	0.91	0.41	27.97	0.20
hsa-miR-376b-3p	2%	0.90	0.39	27.96	0.24
hsa-miR-378a-5p	2%	0.90	0.38	27.96	0.27
hsa-miR-485-3p	2%	0.86	0.56	27.92	0.49
hsa-miR-1255b-5p	2%	0.92	0.38	27.98	0.16
hsa-miR-125a-3p	2%	0.92	0.37	27.98	0.14
hsa-miR-1260a	2%	0.83	0.86	27.89	0.85
hsa-miR-15a-3p	2%	0.92	0.37	27.98	0.16
hsa-miR-214-5p	2%	0.91	0.39	27.97	0.26
hsa-miR-215-5p	2%	0.84	0.83	27.90	0.81
hsa-miR-433-3p	2%	0.90	0.47	27.96	0.31
hsa-miR-489-3p	2%	0.92	0.37	27.98	0.15
hsa-miR-513c-5	2%	0.90	0.44	27.96	0.31
hsa-miR-523-3p	2%	0.83	1.11	27.89	1.04
hsa-miR-551b-3p	2%	0.82	1.25	27.88	1.22
hsa-miR-616-3p	2%	0.91	0.42	27.98	0.20
hsa-miR-628-5p	2%	0.85	0.82	27.91	0.73
hsa-miR-655-3p	2%	0.91	0.38	27.97	0.22
hsa-miR-9-3p	2%	0.91	0.37	27.97	0.22
hsa-miR-943	2%	0.92	0.40	27.98	0.17
hsa-miR-137	2%	0.92	0.37	27.98	0.15
hsa-miR-1201	1%	0.93	0.40	27.99	0.14

hsa-miR-1247-5p	1%	0.89	0.57	27.95	0.54
hsa-miR-1300	1%	0.92	0.38	27.98	0.18
hsa-miR-154-5p	1%	0.92	0.43	27.98	0.24
hsa-miR-183-3p	1%	0.93	0.40	27.99	0.09
hsa-miR-219a-5p	1%	0.93	0.41	27.99	0.11
hsa-miR-302b-3p	1%	0.93	0.40	27.99	0.12
hsa-miR-302c-3p	1%	0.92	0.39	27.98	0.17
hsa-miR-302c-5p	1%	0.92	0.42	27.98	0.17
hsa-miR-331-5p	1%	0.92	0.40	27.98	0.22
hsa-miR-363-3p	1%	0.89	0.58	27.95	0.56
hsa-miR-409-5p	1%	0.93	0.38	27.99	0.09
hsa-miR-455-3p	1%	0.90	0.52	27.96	0.47
hsa-miR-486-3p	1%	0.93	0.37	27.99	0.12
hsa-miR-501-3p	1%	0.93	0.39	27.99	0.16
hsa-miR-509-3-5	1%	0.92	0.44	27.98	0.21
hsa-miR-518d-3p	1%	0.92	0.45	27.98	0.22
hsa-miR-518d-5p	1%	0.93	0.40	27.99	0.12
hsa-miR-520d-3p	1%	0.92	0.42	27.98	0.22
hsa-miR-520f-3p	1%	0.93	0.39	27.99	0.11
hsa-miR-548a-3p	1%	0.93	0.38	27.99	0.11
hsa-miR-614	1%	0.93	0.39	27.99	0.11
hsa-miR-873-5p	1%	0.93	0.37	27.99	0.11
hsa-miR-874-3p	1%	0.91	0.40	27.98	0.28
hsa-miR-890	1%	0.92	0.40	27.99	0.16
hsa-let-7a-3	0%	0.94	0.38	28.00	0.00
hsa-let-7b-3p	0%	0.94	0.38	28.00	0.00
hsa-let-7c#	0%	0.94	0.38	28.00	0.00
hsa-let-7e-3p	0%	0.94	0.38	28.00	0.00
hsa-let-7f-1-3p	0%	0.94	0.38	28.00	0.00
hsa-let-7f-2-3p	0%	0.94	0.38	28.00	0.00
hsa-let-7g-3p	0%	0.94	0.38	28.00	0.00
hsa-let-7i-3p	0%	0.94	0.38	28.00	0.00
hsa-miR-1-3p	0%	0.94	0.38	28.00	0.00
hsa-miR-100-3	0%	0.94	0.38	28.00	0.00
hsa-miR-101-5	0%	0.94	0.38	28.00	0.00
hsa-miR-105-3p	0%	0.94	0.38	28.00	0.00
hsa-miR-105-5p	0%	0.94	0.38	28.00	0.00
hsa-miR-106a-3	0%	0.94	0.38	28.00	0.00
hsa-miR-106b-3p	0%	0.94	0.38	28.00	0.00
hsa-miR-10a-3p	0%	0.94	0.38	28.00	0.00
hsa-miR-1178-3p	0%	0.94	0.38	28.00	0.00
hsa-miR-1179	0%	0.94	0.38	28.00	0.00
hsa-miR-1182	0%	0.94	0.38	28.00	0.00

hsa-miR-1183	0%	0.94	0.38	28.00	0.00
hsa-miR-1184	0%	0.94	0.38	28.00	0.00
hsa-miR-1197	0%	0.94	0.38	28.00	0.00
hsa-miR-1200	0%	0.94	0.38	28.00	0.00
hsa-miR-1203	0%	0.94	0.38	28.00	0.00
hsa-miR-1204	0%	0.94	0.38	28.00	0.00
hsa-miR-1205	0%	0.94	0.38	28.00	0.00
hsa-miR-1206	0%	0.94	0.38	28.00	0.00
hsa-miR-1208	0%	0.94	0.38	28.00	0.00
hsa-miR-122-3p	0%	0.94	0.38	28.00	0.00
hsa-miR-1224-3p	0%	0.94	0.38	28.00	0.00
hsa-miR-1225-3p	0%	0.94	0.38	28.00	0.00
hsa-miR-1228-5	0%	0.94	0.38	28.00	0.00
hsa-miR-1233-3p	0%	0.94	0.38	28.00	0.00
hsa-miR-1236-3p	0%	0.94	0.38	28.00	0.00
hsa-miR-1238-3	0%	0.94	0.38	28.00	0.00
hsa-miR-124	0%	0.94	0.38	28.00	0.00
hsa-miR-1243	0%	0.94	0.38	28.00	0.00
hsa-miR-1245a	0%	0.94	0.38	28.00	0.00
hsa-miR-1248	0%	0.94	0.38	28.00	0.00
hsa-miR-1249-3p	0%	0.94	0.38	28.00	0.00
hsa-miR-1250-5p	0%	0.94	0.38	28.00	0.00
hsa-miR-1251-5p	0%	0.94	0.38	28.00	0.00
hsa-miR-1252-5	0%	0.94	0.38	28.00	0.00
hsa-miR-1253	0%	0.94	0.38	28.00	0.00
hsa-miR-1254	0%	0.94	0.38	28.00	0.00
hsa-miR-1255	0%	0.94	0.38	28.00	0.00
hsa-miR-1256	0%	0.94	0.38	28.00	0.00
hsa-miR-1257	0%	0.94	0.38	28.00	0.00
hsa-miR-1259	0%	0.94	0.38	28.00	0.00
hsa-miR-1262	0%	0.94	0.38	28.00	0.00
hsa-miR-1263	0%	0.94	0.38	28.00	0.00
hsa-miR-1264	0%	0.94	0.38	28.00	0.00
hsa-miR-1265	0%	0.94	0.38	28.00	0.00
hsa-miR-1267	0%	0.94	0.38	28.00	0.00
hsa-miR-1269	0%	0.94	0.38	28.00	0.00
hsa-miR-127	0%	0.94	0.38	28.00	0.00
hsa-miR-1270	0%	0.94	0.38	28.00	0.00
hsa-miR-1272	0%	0.94	0.38	28.00	0.00
hsa-miR-1275	0%	0.94	0.38	28.00	0.00
hsa-miR-1276	0%	0.94	0.38	28.00	0.00
hsa-miR-1282	0%	0.94	0.38	28.00	0.00
hsa-miR-1283	0%	0.94	0.38	28.00	0.00

hsa-miR-1284	0%	0.94	0.38	28.00	0.00
hsa-miR-1285-3p	0%	0.94	0.38	28.00	0.00
hsa-miR-1286	0%	0.94	0.38	28.00	0.00
hsa-miR-1288-3p	0%	0.94	0.38	28.00	0.00
hsa-miR-1289	0%	0.94	0.38	28.00	0.00
hsa-miR-129-1-3p	0%	0.94	0.38	28.00	0.00
hsa-miR-129-2-3p	0%	0.94	0.38	28.00	0.00
hsa-miR-129-5p	0%	0.94	0.38	28.00	0.00
hsa-miR-1292-5p	0%	0.94	0.38	28.00	0.00
hsa-miR-1293	0%	0.94	0.38	28.00	0.00
hsa-miR-1294	0%	0.94	0.38	28.00	0.00
hsa-miR-1296-5p	0%	0.94	0.38	28.00	0.00
hsa-miR-1298-5p	0%	0.94	0.38	28.00	0.00
hsa-miR-130	0%	0.94	0.38	28.00	0.00
hsa-miR-1301-3p	0%	0.94	0.38	28.00	0.00
hsa-miR-1304-5	0%	0.94	0.38	28.00	0.00
hsa-miR-1305	0%	0.94	0.38	28.00	0.00
hsa-miR-130a-5	0%	0.94	0.38	28.00	0.00
hsa-miR-130b-5	0%	0.94	0.38	28.00	0.00
hsa-miR-132-5p	0%	0.94	0.38	28.00	0.00
hsa-miR-1324	0%	0.94	0.38	28.00	0.00
hsa-miR-133b	0%	0.94	0.38	28.00	0.00
hsa-miR-135b-3p	0%	0.94	0.38	28.00	0.00
hsa-miR-136-5p	0%	0.94	0.38	28.00	0.00
hsa-miR-138-2-3	0%	0.94	0.38	28.00	0.00
hsa-miR-138-5p	0%	0.94	0.38	28.00	0.00
hsa-miR-139-3p	0%	0.94	0.38	28.00	0.00
hsa-miR-141-5	0%	0.94	0.38	28.00	0.00
hsa-miR-142-5p	0%	0.94	0.38	28.00	0.00
hsa-miR-143-5p	0%	0.94	0.38	28.00	0.00
hsa-miR-144-3p	0%	0.94	0.38	28.00	0.00
hsa-miR-145-3p	0%	0.94	0.38	28.00	0.00
hsa-miR-146a-3	0%	0.94	0.38	28.00	0.00
hsa-miR-147a	0%	0.94	0.38	28.00	0.00
hsa-miR-147b	0%	0.94	0.38	28.00	0.00
hsa-miR-148a-5p	0%	0.94	0.38	28.00	0.00
hsa-miR-148b-5	0%	0.94	0.38	28.00	0.00
hsa-miR-149-3	0%	0.94	0.38	28.00	0.00
hsa-miR-155	0%	0.94	0.38	28.00	0.00
hsa-miR-155-3	0%	0.94	0.38	28.00	0.00
hsa-miR-15b-3p	0%	0.94	0.38	28.00	0.00
hsa-miR-16-2-3p	0%	0.94	0.38	28.00	0.00
hsa-miR-17-3p	0%	0.94	0.38	28.00	0.00

hsa-miR-181a-3p	0%	0.94	0.38	28.00	0.00
hsa-miR-181c-3	0%	0.94	0.38	28.00	0.00
hsa-miR-182-3p	0%	0.94	0.38	28.00	0.00
hsa-miR-182-5p	0%	0.94	0.38	28.00	0.00
hsa-miR-183-5p	0%	0.94	0.38	28.00	0.00
hsa-miR-185-3	0%	0.94	0.38	28.00	0.00
hsa-miR-186-3	0%	0.94	0.38	28.00	0.00
hsa-miR-188-3p	0%	0.94	0.38	28.00	0.00
hsa-miR-18a-3	0%	0.94	0.38	28.00	0.00
hsa-miR-18b-3	0%	0.94	0.38	28.00	0.00
hsa-miR-18b-5p	0%	0.94	0.38	28.00	0.00
hsa-miR-191-3p	0%	0.94	0.38	28.00	0.00
hsa-miR-192-3p	0%	0.94	0.38	28.00	0.00
hsa-miR-193a-3p	0%	0.94	0.38	28.00	0.00
hsa-miR-194-3p	0%	0.94	0.38	28.00	0.00
hsa-miR-195-3p	0%	0.94	0.38	28.00	0.00
hsa-miR-196a-3	0%	0.94	0.38	28.00	0.00
hsa-miR-196b-5p	0%	0.94	0.38	28.00	0.00
hsa-miR-198	0%	0.94	0.38	28.00	0.00
hsa-miR-19a-5	0%	0.94	0.38	28.00	0.00
hsa-miR-19b-1-5p	0%	0.94	0.38	28.00	0.00
hsa-miR-200a-5p	0%	0.94	0.38	28.00	0.00
hsa-miR-200b-5p	0%	0.94	0.38	28.00	0.00
hsa-miR-200c-5p	0%	0.94	0.38	28.00	0.00
hsa-miR-208a-3p	0%	0.94	0.38	28.00	0.00
hsa-miR-208b-3p	0%	0.94	0.38	28.00	0.00
hsa-miR-20b-3	0%	0.94	0.38	28.00	0.00
hsa-miR-21-3	0%	0.94	0.38	28.00	0.00
hsa-miR-211-5p	0%	0.94	0.38	28.00	0.00
hsa-miR-213	0%	0.94	0.38	28.00	0.00
hsa-miR-216a-5p	0%	0.94	0.38	28.00	0.00
hsa-miR-216b-5p	0%	0.94	0.38	28.00	0.00
hsa-miR-217	0%	0.94	0.38	28.00	0.00
hsa-miR-218-1-3	0%	0.94	0.38	28.00	0.00
hsa-miR-218-2-3p	0%	0.94	0.38	28.00	0.00
hsa-miR-219a-1-3p	0%	0.94	0.38	28.00	0.00
hsa-miR-219a-2-3p	0%	0.94	0.38	28.00	0.00
hsa-miR-22-3p	0%	0.94	0.38	28.00	0.00
hsa-miR-220	0%	0.94	0.38	28.00	0.00
hsa-miR-220b	0%	0.94	0.38	28.00	0.00
hsa-miR-220c	0%	0.94	0.38	28.00	0.00
hsa-miR-221-5p	0%	0.94	0.38	28.00	0.00
hsa-miR-23a-3p	0%	0.94	0.38	28.00	0.00

hsa-miR-23a-5p	0%	0.94	0.38	28.00	0.00
hsa-miR-23b-3p	0%	0.94	0.38	28.00	0.00
hsa-miR-23b-5p	0%	0.94	0.38	28.00	0.00
hsa-miR-24-1-5	0%	0.94	0.38	28.00	0.00
hsa-miR-24-2-5	0%	0.94	0.38	28.00	0.00
hsa-miR-25-5	0%	0.94	0.38	28.00	0.00
hsa-miR-26a-1-3p	0%	0.94	0.38	28.00	0.00
hsa-miR-26a-2-3p	0%	0.94	0.38	28.00	0.00
hsa-miR-26b-3p	0%	0.94	0.38	28.00	0.00
hsa-miR-296-3p	0%	0.94	0.38	28.00	0.00
hsa-miR-298	0%	0.94	0.38	28.00	0.00
hsa-miR-299-3	0%	0.94	0.38	28.00	0.00
hsa-miR-299-5p	0%	0.94	0.38	28.00	0.00
hsa-miR-29b-1-5p	0%	0.94	0.38	28.00	0.00
hsa-miR-29b-2-5p	0%	0.94	0.38	28.00	0.00
hsa-miR-302a-5p	0%	0.94	0.38	28.00	0.00
hsa-miR-302b-5	0%	0.94	0.38	28.00	0.00
hsa-miR-302d-3p	0%	0.94	0.38	28.00	0.00
hsa-miR-302d-5p	0%	0.94	0.38	28.00	0.00
hsa-miR-30b-3p	0%	0.94	0.38	28.00	0.00
hsa-miR-30c-1-3p	0%	0.94	0.38	28.00	0.00
hsa-miR-30c-2-3p	0%	0.94	0.38	28.00	0.00
hsa-miR-32-3p	0%	0.94	0.38	28.00	0.00
hsa-miR-32-5p	0%	0.94	0.38	28.00	0.00
hsa-miR-325	0%	0.94	0.38	28.00	0.00
hsa-miR-326	0%	0.94	0.38	28.00	0.00
hsa-miR-330-5p	0%	0.94	0.38	28.00	0.00
hsa-miR-335-3p	0%	0.94	0.38	28.00	0.00
hsa-miR-337-3p	0%	0.94	0.38	28.00	0.00
hsa-miR-338-3p	0%	0.94	0.38	28.00	0.00
hsa-miR-33a-3p	0%	0.94	0.38	28.00	0.00
hsa-miR-33a-5p	0%	0.94	0.38	28.00	0.00
hsa-miR-33b-5p	0%	0.94	0.38	28.00	0.00
hsa-miR-342-5p	0%	0.94	0.38	28.00	0.00
hsa-miR-346	0%	0.94	0.38	28.00	0.00
hsa-miR-361-3p	0%	0.94	0.38	28.00	0.00
hsa-miR-362-3p	0%	0.94	0.38	28.00	0.00
hsa-miR-363-5p	0%	0.94	0.38	28.00	0.00
hsa-miR-367-3p	0%	0.94	0.38	28.00	0.00
hsa-miR-367-5p	0%	0.94	0.38	28.00	0.00
hsa-miR-369-5p	0%	0.94	0.38	28.00	0.00
hsa-miR-371a-3p	0%	0.94	0.38	28.00	0.00
hsa-miR-374a-3p	0%	0.94	0.38	28.00	0.00

hsa-miR-374b-3	0%	0.94	0.38	28.00	0.00
hsa-miR-376a-5	0%	0.94	0.38	28.00	0.00
hsa-miR-377-3p	0%	0.94	0.38	28.00	0.00
hsa-miR-377-5p	0%	0.94	0.38	28.00	0.00
hsa-miR-380-3p	0%	0.94	0.38	28.00	0.00
hsa-miR-384	0%	0.94	0.38	28.00	0.00
hsa-miR-411-3p	0%	0.94	0.38	28.00	0.00
hsa-miR-412-3p	0%	0.94	0.38	28.00	0.00
hsa-miR-422a	0%	0.94	0.38	28.00	0.00
hsa-miR-425-5p	0%	0.94	0.38	28.00	0.00
hsa-miR-431-3p	0%	0.94	0.38	28.00	0.00
hsa-miR-431-5p	0%	0.94	0.38	28.00	0.00
hsa-miR-432-3	0%	0.94	0.38	28.00	0.00
hsa-miR-448	0%	0.94	0.38	28.00	0.00
hsa-miR-450b-3p	0%	0.94	0.38	28.00	0.00
hsa-miR-450b-5p	0%	0.94	0.38	28.00	0.00
hsa-miR-452-3	0%	0.94	0.38	28.00	0.00
hsa-miR-453	0%	0.94	0.38	28.00	0.00
hsa-miR-454-5	0%	0.94	0.38	28.00	0.00
hsa-miR-485-5p	0%	0.94	0.38	28.00	0.00
hsa-miR-486-5p	0%	0.94	0.38	28.00	0.00
hsa-miR-487a-3p	0%	0.94	0.38	28.00	0.00
hsa-miR-488-3p	0%	0.94	0.38	28.00	0.00
hsa-miR-490-3p	0%	0.94	0.38	28.00	0.00
hsa-miR-491-3p	0%	0.94	0.38	28.00	0.00
hsa-miR-492	0%	0.94	0.38	28.00	0.00
hsa-miR-493-3p	0%	0.94	0.38	28.00	0.00
hsa-miR-497	0%	0.94	0.38	28.00	0.00
hsa-miR-497-3p	0%	0.94	0.38	28.00	0.00
hsa-miR-499a-3p	0%	0.94	0.38	28.00	0.00
hsa-miR-501-5p	0%	0.94	0.38	28.00	0.00
hsa-miR-504-5p	0%	0.94	0.38	28.00	0.00
hsa-miR-505-5	0%	0.94	0.38	28.00	0.00
hsa-miR-507	0%	0.94	0.38	28.00	0.00
hsa-miR-508-5	0%	0.94	0.38	28.00	0.00
hsa-miR-511-5p	0%	0.94	0.38	28.00	0.00
hsa-miR-512-3p	0%	0.94	0.38	28.00	0.00
hsa-miR-512-5	0%	0.94	0.38	28.00	0.00
hsa-miR-513a-5p	0%	0.94	0.38	28.00	0.00
hsa-miR-513b-5p	0%	0.94	0.38	28.00	0.00
hsa-miR-515-3p	0%	0.94	0.38	28.00	0.00
hsa-miR-515-5p	0%	0.94	0.38	28.00	0.00
hsa-miR-516-3p	0%	0.94	0.38	28.00	0.00

hsa-miR-516a-5p	0%	0.94	0.38	28.00	0.00
hsa-miR-516b-5p	0%	0.94	0.38	28.00	0.00
hsa-miR-517-5p	0%	0.94	0.38	28.00	0.00
hsa-miR-517a-3p	0%	0.94	0.38	28.00	0.00
hsa-miR-517b-3p	0%	0.94	0.38	28.00	0.00
hsa-miR-517c-3p	0%	0.94	0.38	28.00	0.00
hsa-miR-518a-3p	0%	0.94	0.38	28.00	0.00
hsa-miR-518a-5p	0%	0.94	0.38	28.00	0.00
hsa-miR-518b	0%	0.94	0.38	28.00	0.00
hsa-miR-518c-3p	0%	0.94	0.38	28.00	0.00
hsa-miR-518c-5	0%	0.94	0.38	28.00	0.00
hsa-miR-518e-3p	0%	0.94	0.38	28.00	0.00
hsa-miR-518e-5p	0%	0.94	0.38	28.00	0.00
hsa-miR-518f-3p	0%	0.94	0.38	28.00	0.00
hsa-miR-518f-5	0%	0.94	0.38	28.00	0.00
hsa-miR-519a-3p	0%	0.94	0.38	28.00	0.00
hsa-miR-519b-3p	0%	0.94	0.38	28.00	0.00
hsa-miR-519c-3p	0%	0.94	0.38	28.00	0.00
hsa-miR-519d-3p	0%	0.94	0.38	28.00	0.00
hsa-miR-519e-3p	0%	0.94	0.38	28.00	0.00
hsa-miR-519e-5	0%	0.94	0.38	28.00	0.00
hsa-miR-520a-3p	0%	0.94	0.38	28.00	0.00
hsa-miR-520a-5p	0%	0.94	0.38	28.00	0.00
hsa-miR-520d-5p	0%	0.94	0.38	28.00	0.00
hsa-miR-520g-3p	0%	0.94	0.38	28.00	0.00
hsa-miR-520h	0%	0.94	0.38	28.00	0.00
hsa-miR-521	0%	0.94	0.38	28.00	0.00
hsa-miR-522-3p	0%	0.94	0.38	28.00	0.00
hsa-miR-524-3p	0%	0.94	0.38	28.00	0.00
hsa-miR-524-5p	0%	0.94	0.38	28.00	0.00
hsa-miR-525-5p	0%	0.94	0.38	28.00	0.00
hsa-miR-525-3p	0%	0.94	0.38	28.00	0.00
hsa-miR-526b-5p	0%	0.94	0.38	28.00	0.00
hsa-miR-541-3	0%	0.94	0.38	28.00	0.00
hsa-miR-541-5p	0%	0.94	0.38	28.00	0.00
hsa-miR-542-5p	0%	0.94	0.38	28.00	0.00
hsa-miR-544a	0%	0.94	0.38	28.00	0.00
hsa-miR-545-3p	0%	0.94	0.38	28.00	0.00
hsa-miR-545-5	0%	0.94	0.38	28.00	0.00
hsa-miR-548a-5	0%	0.94	0.38	28.00	0.00
hsa-miR-548b-3p	0%	0.94	0.38	28.00	0.00
hsa-miR-548c-3p	0%	0.94	0.38	28.00	0.00
hsa-miR-548d-3	0%	0.94	0.38	28.00	0.00

hsa-miR-548e-3	0%	0.94	0.38	28.00	0.00
hsa-miR-548g-3	0%	0.94	0.38	28.00	0.00
hsa-miR-548h-5p	0%	0.94	0.38	28.00	0.00
hsa-miR-548i	0%	0.94	0.38	28.00	0.00
hsa-miR-548j-5	0%	0.94	0.38	28.00	0.00
hsa-miR-548k	0%	0.94	0.38	28.00	0.00
hsa-miR-548l	0%	0.94	0.38	28.00	0.00
hsa-miR-548m	0%	0.94	0.38	28.00	0.00
hsa-miR-548n	0%	0.94	0.38	28.00	0.00
hsa-miR-548p	0%	0.94	0.38	28.00	0.00
hsa-miR-549a	0%	0.94	0.38	28.00	0.00
hsa-miR-550a-3	0%	0.94	0.38	28.00	0.00
hsa-miR-550a-5p	0%	0.94	0.38	28.00	0.00
hsa-miR-551a	0%	0.94	0.38	28.00	0.00
hsa-miR-551b-5	0%	0.94	0.38	28.00	0.00
hsa-miR-552-3p	0%	0.94	0.38	28.00	0.00
hsa-miR-553	0%	0.94	0.38	28.00	0.00
hsa-miR-554	0%	0.94	0.38	28.00	0.00
hsa-miR-555	0%	0.94	0.38	28.00	0.00
hsa-miR-556-3p	0%	0.94	0.38	28.00	0.00
hsa-miR-556-5p	0%	0.94	0.38	28.00	0.00
hsa-miR-557	0%	0.94	0.38	28.00	0.00
hsa-miR-558	0%	0.94	0.38	28.00	0.00
hsa-miR-559	0%	0.94	0.38	28.00	0.00
hsa-miR-561-3	0%	0.94	0.38	28.00	0.00
hsa-miR-562	0%	0.94	0.38	28.00	0.00
hsa-miR-563	0%	0.94	0.38	28.00	0.00
hsa-miR-564	0%	0.94	0.38	28.00	0.00
hsa-miR-566	0%	0.94	0.38	28.00	0.00
hsa-miR-567	0%	0.94	0.38	28.00	0.00
hsa-miR-569	0%	0.94	0.38	28.00	0.00
hsa-miR-570-3	0%	0.94	0.38	28.00	0.00
hsa-miR-571	0%	0.94	0.38	28.00	0.00
hsa-miR-573	0%	0.94	0.38	28.00	0.00
hsa-miR-575	0%	0.94	0.38	28.00	0.00
hsa-miR-576-5	0%	0.94	0.38	28.00	0.00
hsa-miR-577	0%	0.94	0.38	28.00	0.00
hsa-miR-578	0%	0.94	0.38	28.00	0.00
hsa-miR-579-3p	0%	0.94	0.38	28.00	0.00
hsa-miR-580-3p	0%	0.94	0.38	28.00	0.00
hsa-miR-581	0%	0.94	0.38	28.00	0.00
hsa-miR-582-3p	0%	0.94	0.38	28.00	0.00
hsa-miR-582-5p	0%	0.94	0.38	28.00	0.00

hsa-miR-583	0%	0.94	0.38	28.00	0.00
hsa-miR-584-5p	0%	0.94	0.38	28.00	0.00
hsa-miR-585-3	0%	0.94	0.38	28.00	0.00
hsa-miR-586	0%	0.94	0.38	28.00	0.00
hsa-miR-587	0%	0.94	0.38	28.00	0.00
hsa-miR-588	0%	0.94	0.38	28.00	0.00
hsa-miR-589-3	0%	0.94	0.38	28.00	0.00
hsa-miR-589-5p	0%	0.94	0.38	28.00	0.00
hsa-miR-591	0%	0.94	0.38	28.00	0.00
hsa-miR-592	0%	0.94	0.38	28.00	0.00
hsa-miR-593-3p	0%	0.94	0.38	28.00	0.00
hsa-miR-593-5	0%	0.94	0.38	28.00	0.00
hsa-miR-595	0%	0.94	0.38	28.00	0.00
hsa-miR-596	0%	0.94	0.38	28.00	0.00
hsa-miR-599	0%	0.94	0.38	28.00	0.00
hsa-miR-600	0%	0.94	0.38	28.00	0.00
hsa-miR-603	0%	0.94	0.38	28.00	0.00
hsa-miR-604	0%	0.94	0.38	28.00	0.00
hsa-miR-606	0%	0.94	0.38	28.00	0.00
hsa-miR-607	0%	0.94	0.38	28.00	0.00
hsa-miR-608	0%	0.94	0.38	28.00	0.00
hsa-miR-609	0%	0.94	0.38	28.00	0.00
hsa-miR-613	0%	0.94	0.38	28.00	0.00
hsa-miR-615-5p	0%	0.94	0.38	28.00	0.00
hsa-miR-616-5	0%	0.94	0.38	28.00	0.00
hsa-miR-617	0%	0.94	0.38	28.00	0.00
hsa-miR-618	0%	0.94	0.38	28.00	0.00
hsa-miR-620	0%	0.94	0.38	28.00	0.00
hsa-miR-621	0%	0.94	0.38	28.00	0.00
hsa-miR-623	0%	0.94	0.38	28.00	0.00
hsa-miR-624-3p	0%	0.94	0.38	28.00	0.00
hsa-miR-625-5	0%	0.94	0.38	28.00	0.00
hsa-miR-626	0%	0.94	0.38	28.00	0.00
hsa-miR-627-5p	0%	0.94	0.38	28.00	0.00
hsa-miR-628-3p	0%	0.94	0.38	28.00	0.00
hsa-miR-629-5	0%	0.94	0.38	28.00	0.00
hsa-miR-630	0%	0.94	0.38	28.00	0.00
hsa-miR-631	0%	0.94	0.38	28.00	0.00
hsa-miR-633	0%	0.94	0.38	28.00	0.00
hsa-miR-634	0%	0.94	0.38	28.00	0.00
hsa-miR-635	0%	0.94	0.38	28.00	0.00
hsa-miR-637	0%	0.94	0.38	28.00	0.00
hsa-miR-639	0%	0.94	0.38	28.00	0.00

hsa-miR-640	0%	0.94	0.38	28.00	0.00
hsa-miR-641	0%	0.94	0.38	28.00	0.00
hsa-miR-643	0%	0.94	0.38	28.00	0.00
hsa-miR-644	0%	0.94	0.38	28.00	0.00
hsa-miR-646	0%	0.94	0.38	28.00	0.00
hsa-miR-647	0%	0.94	0.38	28.00	0.00
hsa-miR-648	0%	0.94	0.38	28.00	0.00
hsa-miR-649	0%	0.94	0.38	28.00	0.00
hsa-miR-650	0%	0.94	0.38	28.00	0.00
hsa-miR-651-5	0%	0.94	0.38	28.00	0.00
hsa-miR-653-5p	0%	0.94	0.38	28.00	0.00
hsa-miR-654-3p	0%	0.94	0.38	28.00	0.00
hsa-miR-654-5p	0%	0.94	0.38	28.00	0.00
hsa-miR-656-3p	0%	0.94	0.38	28.00	0.00
hsa-miR-657	0%	0.94	0.38	28.00	0.00
hsa-miR-658	0%	0.94	0.38	28.00	0.00
hsa-miR-661	0%	0.94	0.38	28.00	0.00
hsa-miR-662	0%	0.94	0.38	28.00	0.00
hsa-miR-664a-3p	0%	0.94	0.38	28.00	0.00
hsa-miR-665	0%	0.94	0.38	28.00	0.00
hsa-miR-668-3p	0%	0.94	0.38	28.00	0.00
hsa-miR-672	0%	0.94	0.38	28.00	0.00
hsa-miR-674	0%	0.94	0.38	28.00	0.00
hsa-miR-675-5p	0%	0.94	0.38	28.00	0.00
hsa-miR-7-2-3	0%	0.94	0.38	28.00	0.00
hsa-miR-708-3p	0%	0.94	0.38	28.00	0.00
hsa-miR-765	0%	0.94	0.38	28.00	0.00
hsa-miR-767-3p	0%	0.94	0.38	28.00	0.00
hsa-miR-767-5p	0%	0.94	0.38	28.00	0.00
hsa-miR-769-3	0%	0.94	0.38	28.00	0.00
hsa-miR-770-5p	0%	0.94	0.38	28.00	0.00
hsa-miR-802	0%	0.94	0.38	28.00	0.00
hsa-miR-871	0%	0.94	0.38	28.00	0.00
hsa-miR-872	0%	0.94	0.38	28.00	0.00
hsa-miR-875-3	0%	0.94	0.38	28.00	0.00
hsa-miR-875-5p	0%	0.94	0.38	28.00	0.00
hsa-miR-876-3p	0%	0.94	0.38	28.00	0.00
hsa-miR-876-5p	0%	0.94	0.38	28.00	0.00
hsa-miR-885-3p	0%	0.94	0.38	28.00	0.00
hsa-miR-887-3p	0%	0.94	0.38	28.00	0.00
hsa-miR-888-3	0%	0.94	0.38	28.00	0.00
hsa-miR-891a-5	0%	0.94	0.38	28.00	0.00
hsa-miR-891b	0%	0.94	0.38	28.00	0.00

hsa-miR-892a	0%	0.94	0.38	28.00	0.00
hsa-miR-892b	0%	0.94	0.38	28.00	0.00
hsa-miR-920	0%	0.94	0.38	28.00	0.00
hsa-miR-921	0%	0.94	0.38	28.00	0.00
hsa-miR-922	0%	0.94	0.38	28.00	0.00
hsa-miR-924	0%	0.94	0.38	28.00	0.00
hsa-miR-92a-1-5p	0%	0.94	0.38	28.00	0.00
hsa-miR-92a-2-5	0%	0.94	0.38	28.00	0.00
hsa-miR-92b-5	0%	0.94	0.38	28.00	0.00
hsa-miR-933	0%	0.94	0.38	28.00	0.00
hsa-miR-934	0%	0.94	0.38	28.00	0.00
hsa-miR-935	0%	0.94	0.38	28.00	0.00
hsa-miR-936	0%	0.94	0.38	28.00	0.00
hsa-miR-937-3p	0%	0.94	0.38	28.00	0.00
hsa-miR-938	0%	0.94	0.38	28.00	0.00
hsa-miR-939-5p	0%	0.94	0.38	28.00	0.00
hsa-miR-941	0%	0.94	0.38	28.00	0.00
hsa-miR-944	0%	0.94	0.38	28.00	0.00
hsa-miR-96-3	0%	0.94	0.38	28.00	0.00
hsa-miR-96-5p	0%	0.94	0.38	28.00	0.00
mmu-miR-129-3p	0%	0.94	0.38	28.00	0.00
mmu-miR-153	0%	0.94	0.38	28.00	0.00
mmu-miR-187	0%	0.94	0.38	28.00	0.00
mmu-miR-496	0%	0.94	0.38	28.00	0.00
mmu-miR-499	0%	0.94	0.38	28.00	0.00
mmu-miR-615	0%	0.94	0.38	28.00	0.00
mmu-miR-96	0%	0.94	0.38	28.00	0.00
rno-miR-29c	0%	0.94	0.38	28.00	0.00
** Those EV-miRNAs that had a Ct value greater than 28 were coded as 28 (unexpressed)					
^ Normalized Ct values were normalized using the global mean.					

Supplemental Table S2: Fold-change analysis of day one fertilization

EV-miRNA name	Fold Change	p-value
hsa-miR-192	1.80	0.01
hsa-miR-542-3p	1.34	0.02
hsa-miR-29a	1.28	0.03
hsa-let-7c	1.49	0.03
hsa-miR-92a	1.52	0.03
hsa-miR-503	1.50	0.03
hsa-miR-122	-1.47	0.04
hsa-miR-31-3p	1.47	0.04
hsa-miR-210	3.79	0.04
hsa-miR-98	1.71	0.04
hsa-miR-130b	1.65	0.05
hsa-miR-22	1.37	0.05
hsa-miR-20a	1.34	0.06
hsa-miR-539	-1.12	0.06
hsa-miR-28	1.41	0.06
hsa-miR-19a	1.62	0.06
hsa-miR-17	1.33	0.07
hsa-miR-885-5p	-1.41	0.07
hsa-miR-320B	1.45	0.07
hsa-miR-184	1.27	0.08
hsa-miR-301	1.40	0.08
hsa-miR-99b	-1.34	0.08
hsa-miR-532	1.52	0.08
hsa-miR-15b	1.33	0.09
hsa-miR-328	1.40	0.09
hsa-miR-146a	1.72	0.09
rno-miR-7	1.91	0.09
hsa-miR-181a	1.48	0.09
hsa-miR-106a	1.30	0.09
hsa-miR-132	1.53	0.09
hsa-miR-652	1.37	0.10
hsa-miR-127	1.47	0.10
hsa-miR-16	1.40	0.11
hsa-let-7a	3.07	0.11
hsa-miR-193b-5p	1.36	0.11
hsa-miR-28-3p	1.79	0.12
hsa-miR-345	1.27	0.12
hsa-miR-21	2.18	0.12

hsa-miR-26b	1.41	0.12
hsa-miR-202-3p	1.45	0.13
hsa-miR-152	1.49	0.13
hsa-miR-30a-3p	1.49	0.14
hsa-miR-320	1.37	0.14
hsa-miR-130a	1.37	0.14
hsa-miR-454	1.57	0.15
hsa-miR-128a	1.24	0.15
hsa-miR-25	1.41	0.15
hsa-miR-29a-5p	1.48	0.16
hsa-miR-424-3p	1.33	0.16
hsa-miR-15a	1.24	0.16
mmu-miR-140	1.37	0.18
hsa-miR-212	1.27	0.18
hsa-miR-365	1.47	0.19
hsa-miR-194	1.34	0.19
hsa-miR-106b	1.22	0.19
hsa-miR-19b	1.17	0.19
hsa-miR-99b-3p	1.35	0.19
hsa-miR-497	1.22	0.19
hsa-miR-411	1.29	0.20
hsa-miR-141	-1.18	0.20
hsa-let-7d	1.28	0.21
hsa-miR-10b-3p	-1.32	0.21
mmu-miR-495	1.43	0.21
hsa-miR-339-5p	1.93	0.22
hsa-miR-548b-5p	-1.02	0.22
hsa-miR-148a	1.33	0.23
hsa-miR-18a	1.30	0.25
hsa-miR-103	1.23	0.25
hsa-miR-30e-3p	1.32	0.26
hsa-miR-1274A	1.27	0.27
hsa-miR-331	1.20	0.28
hsa-miR-27a	1.30	0.28
hsa-miR-204	1.43	0.28
hsa-miR-145	1.50	0.29
hsa-miR-34a-3p	1.50	0.29
hsa-miR-376c	1.48	0.29
hsa-miR-508	1.23	0.29
hsa-miR-886-5p	1.28	0.30
hsa-miR-26a	1.28	0.31
hsa-miR-423-5p	1.31	0.31

hsa-miR-424	1.06	0.32
hsa-miR-597	-1.18	0.32
hsa-miR-186	1.35	0.33
hsa-miR-886-3p	1.24	0.33
hsa-miR-590-5p	1.25	0.33
hsa-miR-324-5p	1.25	0.34
hsa-miR-10b-5p	-1.21	0.35
hsa-miR-223	1.32	0.35
hsa-miR-30c	1.48	0.35
mmu-miR-491	1.21	0.35
hsa-miR-151-3p	1.20	0.36
hsa-miR-1290	-1.18	0.36
mmu-miR-93	1.31	0.36
hsa-let-7e	1.88	0.37
hsa-miR-1274B	1.14	0.37
hsa-miR-30b	1.31	0.37
hsa-miR-195	1.20	0.38
hsa-miR-125a-5p	1.23	0.38
hsa-miR-410	1.20	0.38
hsa-miR-200b	-1.14	0.40
hsa-miR-484	1.36	0.40
hsa-miR-199a	-1.11	0.41
hsa-miR-374	1.17	0.41
hsa-miR-193a-5p	-1.07	0.41
hsa-miR-744	1.20	0.41
hsa-miR-296	1.33	0.42
hsa-miR-142-3p	1.30	0.44
hsa-miR-375	1.16	0.44
hsa-miR-449b	-1.08	0.44
hsa-miR-203	1.16	0.44
mmu-miR-374-5p	1.20	0.46
hsa-let-7g	1.18	0.46
hsa-miR-10a	-1.06	0.47
hsa-miR-93	1.09	0.47
hsa-miR-483-5p	1.18	0.48
hsa-miR-199a-3p	1.34	0.50
hsa-miR-30a-5p	1.18	0.50
hsa-miR-548c-5p	-1.03	0.50
hsa-miR-1303	-1.05	0.51
hsa-miR-574-3p	1.19	0.51
hsa-miR-27b	1.12	0.52
hsa-miR-29b	1.17	0.53

hsa-miR-509-5p	1.15	0.54
hsa-miR-125b	1.15	0.54
hsa-miR-191	1.12	0.55
hsa-miR-324-3p	1.15	0.55
hsa-miR-629	-1.05	0.56
hsa-miR-135a	1.00	0.56
hsa-miR-24	1.12	0.59
hsa-miR-133a	-1.05	0.59
hsa-miR-139-5p	1.05	0.59
hsa-miR-638	-1.13	0.60
mmu-miR-134	1.16	0.61
hsa-miR-150	-1.08	0.63
mmu-miR-379	1.18	0.63
hsa-miR-155	1.06	0.65
hsa-miR-645	-1.05	0.65
hsa-miR-942	1.10	0.65
hsa-miR-888	-1.26	0.65
hsa-miR-708	1.10	0.67
hsa-miR-1226-5p	1.17	0.68
hsa-miR-335	-1.10	0.68
hsa-miR-221	1.33	0.69
hsa-miR-1291	1.03	0.69
hsa-miR-193b	1.21	0.70
hsa-miR-323-3p	1.14	0.71
hsa-miR-339-3p	1.05	0.71
hsa-miR-1180	1.03	0.71
hsa-miR-532-3p	1.06	0.73
hsa-miR-34a	1.06	0.73
hsa-miR-202	1.10	0.74
hsa-miR-34b	1.02	0.74
hsa-miR-370	1.35	0.74
hsa-miR-99a	1.14	0.75
mmu-miR-451	1.13	0.76
hsa-miR-660	1.07	0.76
hsa-miR-20b	1.10	0.77
hsa-miR-148b	1.08	0.78
hsa-miR-601	1.07	0.78
hsa-miR-222	1.18	0.78
hsa-miR-483-3p	1.08	0.78
hsa-miR-95	1.01	0.79
hsa-miR-769-5p	1.10	0.80
hsa-miR-455	1.03	0.82

hsa-miR-149	1.24	0.82
hsa-miR-146b	1.13	0.84
hsa-miR-214	1.21	0.85
hsa-miR-361	1.05	0.85
hsa-miR-126	-1.03	0.85
hsa-miR-218	1.10	0.85
hsa-miR-143	-1.01	0.87
hsa-miR-30d	-1.02	0.87
hsa-miR-31	1.06	0.89
hsa-miR-572	1.10	0.90
hsa-miR-720	1.19	0.90
hsa-miR-29c	-1.08	0.92
hsa-miR-381	1.01	0.92
hsa-miR-342-3p	1.10	0.92
hsa-miR-766	1.14	0.92
hsa-miR-101	1.05	0.94
hsa-miR-144	1.14	0.95
hsa-miR-598	1.01	0.96
hsa-miR-190	1.11	0.96
hsa-miR-34c	1.08	0.96
hsa-miR-543	-1.01	0.97
hsa-miR-126-5p	1.03	0.98
hsa-miR-205	1.00	0.99
hsa-miR-487b	1.04	0.99
hsa-miR-376a	1.00	0.99
hsa-miR-100	-1.02	1.00
hsa-miR-409-3p	1.00	1.00
hsa-miR-185	-1.01	1.00

Supplemental Table S3: Fold-change analysis of day three embryo quality

EV-miRNA name	Fold Change	p-value
hsa-miR-214	-3.04	0.01
hsa-miR-145	-2.44	0.03
hsa-miR-454	-2.43	0.03
hsa-miR-888	2.08	0.05
hsa-miR-146a	-1.77	0.07
hsa-miR-202	-1.36	0.07
hsa-miR-19b	-1.87	0.08
hsa-miR-18a	-1.43	0.08
hsa-miR-193b	-1.75	0.08
hsa-miR-193a-5p	-1.43	0.10
hsa-miR-708	-1.60	0.11
mmu-miR-93	-1.65	0.11
hsa-miR-597	-1.31	0.12
hsa-miR-132	-1.39	0.12
hsa-miR-126-5p	-1.68	0.13
hsa-miR-483-3p	1.63	0.14
hsa-miR-16	-1.40	0.14
hsa-miR-194	-1.22	0.14
hsa-miR-148b	-1.23	0.15
hsa-miR-143	-1.57	0.15
hsa-miR-128a	-1.20	0.17
hsa-miR-1291	-1.31	0.17
hsa-miR-93	1.09	0.18
hsa-miR-99a	-1.90	0.18
hsa-miR-29c	-1.56	0.18
hsa-miR-324-3p	-1.31	0.19
hsa-miR-574-3p	-1.47	0.19
hsa-miR-142-3p	-1.47	0.22
hsa-miR-205	-1.40	0.22
hsa-miR-24	-1.60	0.22
hsa-miR-320B	-1.29	0.22
hsa-miR-127	-1.26	0.22
hsa-miR-342-3p	-1.30	0.23
hsa-miR-144	-1.52	0.23
hsa-miR-21	-2.06	0.23
hsa-miR-22	-1.22	0.23
hsa-miR-652	1.07	0.24

hsa-miR-193b-5p	-1.23	0.24
hsa-miR-190	-1.20	0.24
hsa-miR-152	-1.34	0.24
hsa-miR-202-3p	-1.20	0.25
hsa-miR-328	-1.26	0.25
hsa-let-7c	-1.34	0.26
hsa-let-7a	-2.30	0.26
mmu-miR-495	-1.31	0.26
hsa-miR-409-3p	-1.56	0.27
hsa-miR-27a	-1.33	0.27
hsa-miR-223	-1.37	0.27
hsa-miR-19a	-1.29	0.28
hsa-miR-20a	-1.22	0.28
hsa-miR-660	-1.23	0.28
mmu-miR-140	-1.27	0.28
hsa-miR-548b-5p	-1.14	0.29
hsa-miR-92a	-1.21	0.29
hsa-miR-106a	-1.22	0.30
hsa-miR-28-3p	-1.49	0.30
hsa-let-7g	-1.28	0.31
hsa-miR-17	-1.23	0.31
hsa-miR-27b	-1.29	0.31
hsa-miR-25	-1.26	0.31
hsa-miR-100	-1.43	0.32
hsa-miR-320	-1.23	0.32
hsa-miR-125a-5p	-1.28	0.32
hsa-miR-766	1.12	0.32
hsa-miR-296	-1.43	0.34
hsa-miR-423-5p	-1.34	0.34
hsa-miR-199a-3p	-1.32	0.34
hsa-miR-222	-1.58	0.34
hsa-miR-324-5p	-1.22	0.34
hsa-miR-10a	-1.25	0.35
hsa-miR-146b	-1.35	0.35
hsa-miR-130a	-1.26	0.35
hsa-miR-532	-1.28	0.35
hsa-miR-29a-5p	-1.25	0.36
hsa-miR-381	1.10	0.36
hsa-let-7d	-1.19	0.36
hsa-miR-186	-1.25	0.37
hsa-let-7e	-1.35	0.37
hsa-miR-28	-1.15	0.37

hsa-miR-331	-1.16	0.38
hsa-miR-212	-1.22	0.38
hsa-miR-195	-1.22	0.38
hsa-miR-15a	-1.15	0.39
hsa-miR-30e-3p	-1.43	0.39
hsa-miR-101	-1.04	0.40
hsa-miR-15b	-1.18	0.40
hsa-miR-221	-1.32	0.42
hsa-miR-122	1.16	0.43
hsa-miR-31-3p	-1.18	0.43
hsa-miR-508	-1.15	0.44
hsa-miR-497	-1.09	0.44
mmu-miR-451	-1.28	0.45
hsa-miR-484	-1.20	0.45
hsa-miR-532-3p	-1.19	0.45
hsa-miR-543	1.08	0.46
hsa-miR-99b-3p	-1.16	0.46
hsa-miR-10b-5p	-1.16	0.46
hsa-miR-483-5p	-1.14	0.46
hsa-miR-942	-1.14	0.46
hsa-miR-376c	-1.23	0.46
hsa-miR-135a	-1.18	0.47
hsa-miR-424-3p	-1.18	0.47
hsa-miR-539	-1.07	0.48
hsa-miR-509-5p	-1.22	0.48
hsa-miR-503	-1.12	0.49
hsa-miR-185	-1.11	0.49
hsa-miR-10b-3p	-1.29	0.50
hsa-miR-34c	1.03	0.50
hsa-miR-95	-1.14	0.50
hsa-miR-130b	-1.09	0.51
hsa-miR-30c	-1.17	0.51
hsa-miR-26b	-1.14	0.51
hsa-miR-106b	-1.12	0.52
hsa-miR-30a-5p	-1.15	0.52
hsa-miR-191	1.01	0.53
hsa-miR-155	1.02	0.54
hsa-miR-204	-1.24	0.54
hsa-miR-629	-1.10	0.54
hsa-miR-601	1.06	0.54
hsa-miR-361	-1.17	0.55
hsa-miR-150	-1.31	0.55

hsa-miR-30a-3p	-1.29	0.55
hsa-miR-30d	-1.23	0.55
rno-miR-7	-1.30	0.55
hsa-miR-218	-1.11	0.56
hsa-miR-590-5p	-1.15	0.56
hsa-miR-29a	-1.07	0.57
hsa-miR-638	1.15	0.57
hsa-miR-345	-1.21	0.59
hsa-miR-99b	1.09	0.59
hsa-miR-374	-1.22	0.60
hsa-miR-301	-1.12	0.61
hsa-miR-375	-1.22	0.61
hsa-miR-34a	-1.10	0.61
hsa-miR-30b	-1.09	0.61
hsa-miR-424	-1.03	0.61
hsa-miR-181a	-1.13	0.62
hsa-miR-199a	-1.15	0.63
hsa-miR-376a	-1.12	0.63
mmu-miR-379	1.03	0.64
hsa-miR-200b	-1.12	0.65
hsa-miR-34a-3p	1.28	0.65
hsa-miR-339-5p	-1.38	0.66
hsa-miR-365	-1.20	0.66
hsa-miR-598	1.05	0.66
hsa-miR-410	-1.09	0.67
hsa-miR-26a	-1.09	0.67
hsa-miR-29b	-1.09	0.69
hsa-miR-323-3p	1.05	0.70
hsa-miR-1180	1.04	0.71
hsa-miR-1226-5p	-1.12	0.71
hsa-miR-31	1.06	0.72
hsa-miR-572	1.48	0.72
hsa-miR-886-3p	1.21	0.73
hsa-miR-1303	-1.04	0.74
hsa-miR-141	-1.11	0.75
hsa-miR-885-5p	1.10	0.75
hsa-miR-203	-1.01	0.75
hsa-miR-34b	1.01	0.75
hsa-miR-149	-1.23	0.77
hsa-miR-20b	-1.16	0.77
hsa-miR-645	1.05	0.77
hsa-miR-744	1.02	0.78

mmu-miR-491	1.05	0.78
mmu-miR-134	-1.06	0.78
hsa-miR-720	1.04	0.79
hsa-miR-98	-1.09	0.81
hsa-miR-1274B	-1.02	0.81
hsa-miR-151-3p	1.04	0.83
hsa-miR-184	1.07	0.84
hsa-miR-210	-1.53	0.84
hsa-miR-339-3p	-1.04	0.85
hsa-miR-487b	1.01	0.85
hsa-miR-125b	-1.05	0.85
hsa-miR-103	-1.00	0.86
hsa-miR-139-5p	-1.05	0.86
hsa-miR-335	-1.04	0.87
hsa-miR-449b	-1.10	0.88
hsa-miR-411	-1.02	0.89
hsa-miR-1290	-1.11	0.90
mmu-miR-374-5p	-1.10	0.91
hsa-miR-133a	-1.12	0.93
hsa-miR-548c-5p	1.01	0.94
hsa-miR-886-5p	1.06	0.94
hsa-miR-192	-1.01	0.96
hsa-miR-455	-1.07	0.96
hsa-miR-1274A	-1.11	0.99
hsa-miR-126	-1.09	1.00
hsa-miR-148a	1.05	1.00
hsa-miR-370	-1.25	1.00
hsa-miR-542-3p	-1.01	1.00
hsa-miR-769-5p	-1.05	1.00

Supplementary Table S4: Expression of EV-miRNA in more than 15% of the FF samples		
EV-miRNA Name	Martinez et al. (N=126)	Machtinger et al., 2016 (N=40)
hsa-let-7a	26%	not detected
hsa-let-7c	99%	not detected
hsa-let-7d	83%	not detected
hsa-let-7e	60%	not detected
hsa-let-7g	81%	not detected
hsa-miR-7-3p	not detected	83%
hsa-miR-9-3p	not detected	23%
hsa-miR-10a	52%	not detected
hsa-miR-10b-3p	51%	90%
hsa-miR-10b-5p	25%	not detected
hsa-miR-15a	61%	not detected
hsa-miR-15b	98%	not detected
hsa-miR-16-1-3p	100%	48%
hsa-miR-16-5p	not detected	45%
hsa-miR-17	100%	not detected
hsa-miR-18a-3p	24%	48%
hsa-miR-19a	91%	not detected
hsa-miR-19b-3p	98%	30%
hsa-miR-19b-1-5p	not detected	20%
hsa-miR-20a	100%	55%
hsa-miR-20b	42%	not detected
hsa-miR-21	87%	25%
hsa-miR-22	49%	53%
hsa-miR-24-3p	98%	15%
hsa-miR-25	98%	not detected
hsa-miR-26a	100%	53%
hsa-miR-26b	99%	20%
hsa-miR-27a	58%	58%
hsa-miR-27b	90%	33%
hsa-miR-28	100%	not detected
hsa-miR-28-3p	75%	not detected
hsa-miR-296	66%	not detected
hsa-miR-29a	36%	not detected
hsa-miR-29a-5p	95%	33%
hsa-miR-29b	65%	not detected
hsa-miR-29c	88%	45%
hsa-miR-30a-3p	89%	48%
hsa-miR-30a-5p	100%	75%

hsa-miR-30b	100%	not detected
hsa-miR-30c	98%	not detected
hsa-miR-30d-5p	98%	100%
hsa-miR-30d-3p		23%
hsa-miR-30e-3p	89%	40%
hsa-miR-31	78%	not detected
hsa-miR-31-3p	95%	73%
hsa-miR-33a-3p	not detected	not detected
hsa-miR-34a	60%	55%
hsa-miR-34a-3p	75%	28%
hsa-miR-34b-3p	18%	48%
hsa-miR-34c	16%	not detected
hsa-miR-92a-3p	100%	not detected
hsa-miR-92a-1-5p	not detected	15%
hsa-miR-93	17%	63%
hsa-miR-95	56%	not detected
hsa-miR-98	18%	not detected
hsa-miR-99a	94%	63%
hsa-miR-99b	25%	not detected
hsa-miR-99b-3p	100%	43%
hsa-miR-100	82%	not detected
hsa-miR-101	27%	not detected
hsa-miR-103	94%	not detected
hsa-miR-106a	100%	not detected
hsa-miR-106b	100%	not detected
hsa-miR-122	19%	not detected
hsa-miR-125a-5p	52%	not detected
hsa-miR-125b-1-3p	98%	25%
hsa-miR-126	78%	not detected
hsa-miR-126-5p	89%	75%
hsa-miR-127	99%	not detected
hsa-miR-128a	53%	not detected
hsa-miR-130a	99%	not detected
hsa-miR-130b-3p	95%	not detected
hsa-miR-132-3p	100%	20%
hsa-miR-133a	34%	not detected
hsa-miR-135a	42%	not detected
hsa-miR-136-3p	not detected	78%
hsa-miR-139-5p	73%	not detected
hsa-miR-141	22%	not detected

hsa-miR-142-3p	89%	not detected
hsa-miR-143	75%	not detected
hsa-miR-144	19%	63%
hsa-miR-145	64%	33%
hsa-miR-146a	94%	not detected
hsa-miR-146b	99%	not detected
hsa-miR-148a	79%	not detected
hsa-miR-148b	44%	38%
hsa-miR-149	37%	not detected
hsa-miR-150	97%	not detected
hsa-miR-151a-5p	not detected	48%
hsa-miR-151-3p	49%	53%
hsa-miR-152	90%	not detected
hsa-miR-155	17%	not detected
hsa-miR-181a	89%	48%
hsa-miR-184	54%	not detected
hsa-miR-185	33%	not detected
hsa-miR-186	88%	not detected
hsa-miR-190b	25%	20%
hsa-miR-191	99%	15%
hsa-miR-192	92%	not detected
hsa-miR-193a-5p	56%	not detected
hsa-miR-193b	54%	not detected
hsa-miR-193b-3p	not detected	23%
hsa-miR-193b-5p	100%	38%
hsa-miR-194	37%	not detected
hsa-miR-195	99%	not detected
hsa-miR-199a	15%	not detected
hsa-miR-199a-3p	20%	not detected
hsa-miR-200b	39%	not detected
hsa-miR-202-5p	25%	68%
hsa-miR-202-3p	98%	not detected
hsa-miR-203	100%	not detected
hsa-miR-204	87%	not detected
hsa-miR-205	55%	not detected
hsa-miR-206	not detected	68%
hsa-miR-210	69%	not detected
hsa-miR-212	100%	not detected
hsa-miR-214	41%	`not detected
hsa-miR-218	100%	not detected

hsa-miR-221	69%	not detected
hsa-miR-222	88%	15%
hsa-miR-223-3p	100%	28%
hsa-miR-301	79%	not detected
hsa-miR-320a	100%	15%
hsa-miR-320b	77%	98%
hsa-miR-323-3p	46%	not detected
hsa-miR-324-3p	73%	not detected
hsa-miR-324-5p	94%	not detected
hsa-miR-328	100%	not detected
hsa-miR-331	100%	not detected
hsa-miR-335	93%	not detected
hsa-miR-338-5p	not detected	23%
hsa-miR-339-3p	23%	not detected
hsa-miR-339-5p	23%	not detected
hsa-miR-340-3p	not detected	63%
hsa-miR-342-3p	23%	not detected
hsa-miR-345	58%	not detected
hsa-miR-361	79%	not detected
hsa-miR-365	77%	not detected
hsa-miR-370	15%	not detected
hsa-miR-374	98%	not detected
hsa-miR-375	91%	not detected
hsa-miR-376a	95%	not detected
hsa-miR-376c	87%	not detected
hsa-miR-378a-3p	not detected	50%
hsa-miR-380-5p	not detected	78%
hsa-miR-381	21%	not detected
hsa-miR-409-3p	79%	60%
hsa-miR-410	78%	not detected
hsa-miR-411	90%	not detected
hsa-miR-423-5p	71%	not detected
hsa-miR-424	95%	not detected
hsa-miR-424-3p	94%	70%
hsa-miR-425-3p	not detected	40%
hsa-miR-449b	17%	not detected
hsa-miR-454-5p	not detected	38%
has-miR-454-3p	49%	not detected
hsa-miR-455	30%	not detected
hsa-miR-483-3p	57%	not detected

hsa-miR-483-5p	100%	not detected
hsa-miR-484	98%	not detected
hsa-miR-487b	17%	not detected
hsa-miR-497-5p	36%	53%
hsa-miR-503	94%	not detected
hsa-miR-505-5p	not detected	65%
hsa-miR-508	57%	not detected
hsa-miR-509-5p	71%	not detected
hsa-miR-520d-3p	not detected	28%
hsa-miR-520f-3p	not detected	40%
hsa-miR-532	85%	not detected
hsa-miR-532-3p	55%	not detected
hsa-miR-539	22%	not detected
hsa-miR-542-3p	40%	not detected
hsa-miR-543	48%	28%
hsa-miR-548b-5p	30%	not detected
hsa-miR-548c-5p	15%	not detected
hsa-miR-550a-5p	not detected	23%
hsa-miR-564	not detected	33%
hsa-miR-572	98%	83%
hsa-miR-574-3p	94%	not detected
hsa-miR-590-5p	95%	not detected
has-miR-454-3p	not detected	70%
hsa-miR-592	not detected	25%
hsa-miR-597	62%	not detected
hsa-miR-598	28%	not detected
hsa-miR-601	48%	68%
hsa-miR-603	not detected	35%
hsa-miR-604	not detected	15%
hsa-miR-616-5p	not detected	23%
hsa-miR-629	55%	73%
hsa-miR-638	72%	20%
hsa-miR-645	78%	55%
hsa-miR-649	not detected	38%
hsa-miR-652	23%	not detected
hsa-miR-660	90%	not detected
has-miR-663B	not detected	75%
hsa-miR-708	60%	not detected
hsa-miR-720	99%	85%
hsa-miR-744	60%	43%

hsa-miR-766	18%	43%
hsa-miR-769-5p	25%	not detected
hsa-miR-885-5p	52%	not detected
hsa-miR-886-3p	99%	not detected
hsa-miR-886-5p	87%	not detected
hsa-miR-888	18%	not detected
hsa-miR-942	85%	80%
hsa-miR-1179	not detected	18%
hsa-miR-1180	32%	45%
hsa-miR-1226-5p	25%	78%
hsa-miR-1227-3p	not detected	28%
hsa-miR-1244	not detected	55%
hsa-miR-1255b-5p	not detected	78%
hsa-miR-1274A	97%	53%
hsa-miR-1274B	100%	50%
hsa-miR-1276	not detected	40%
hsa-miR-1290	50%	65%
hsa-miR-1291	78%	88%
hsa-miR-1300	not detected	70%
hsa-miR-1303	33%	63%
mmu-miR-140	86%	not detected
mmu-miR-374-5p	89%	not detected
mmu-miR-379	46%	not detected
mmu-miR-451	98%	not detected
mmu-miR-491	37%	not detected
mmu-miR-495	45%	not detected
mmu-miR-93	72%	not detected
rno-miR-7	37%	not detected
